# Boosting the Dopant Accommodation in Charge-Transfer Supramolecular Electrets Using Carbohydrate Block Copolymers in Phototransistor Memory

**DOI:** 10.1021/acsomega.6c07010

**Published:** 2026-08-17

**Authors:** Ching-Wei Yang, Qi-An Hong, Yi-Hsun Weng, Ping-Jui Yu, Bi-Hsuan Lin, Cheng-Liang Liu, Yan-Cheng Lin, Wen-Chang Chen

**Affiliations:** † Institute of Polymer Science and Engineering, 33561 National Taiwan University, Taipei 10617, Taiwan; ‡ Department of Chemical Engineering, National Taiwan University, Taipei 10617, Taiwan; § National Synchrotron Radiation Research Center, Hsinchu 300092, Taiwan; ∥ Department of Materials Science and Engineering, National Taiwan University, Taipei 10617, Taiwan; ⊥ Advanced Research Center of Green Materials Science and Technology, National Taiwan University, Taipei 10617, Taiwan; # Department of Chemical Engineering, 34912 National Cheng Kung University, Tainan 70101, Taiwan

## Abstract

Charge-transfer (CT)-based supramolecular systems have attracted considerable interest as electret materials for organic memory devices. However, uncontrolled dopant distribution, limited host–guest compatibility, and the formation of leakage pathways often compromise their morphological stability and memory performance. In this work, carbohydrate-based block copolymers (BCPs), maltotriose-*block*-poly­(1-pyrenemethyl methacrylate) (PPy) and maltotriose-*block*-poly­(benzyl methacrylate) (PB), are employed as supramolecular hosts for incorporating the electron acceptor F_4_-TCNQ, forming a series of supramolecular electret systems for organic phototransistor memory applications. Photoluminescence and time-resolved photoluminescence characterizations confirm efficient photoinduced intermolecular CT interactions in the PPy/F_4_-TCNQ systems, as evidenced by significant fluorescence quenching and a shortened excited-state lifetime. More importantly, solvent-vapor annealing induces supramolecular reorganization within the electret layer, thereby effectively regulating dopant distribution and reducing leakage pathways. Meanwhile, optical, morphological, and surface-energy analyses further reveal that the maltotriose carbohydrate block plays a critical role in suppressing channel interference and stabilizing the supramolecular architecture. As a result, the optimized phototransistor memory exhibits a high photosensitivity of 1.27 × 10^7^ and a memory ratio of 1.61 × 10^6^. Furthermore, reliable memory operation is maintained under low-light-intensity (0.01 mW cm^–2^) and low-operating-voltage (−0.1 V) conditions. These findings demonstrate that carbohydrate-based BCPs provide an effective platform for integrating CT supramolecular interactions with controllable dopant organization, offering a versatile molecular design strategy for developing high-performance organic photomemory devices.

## Graphical abstract

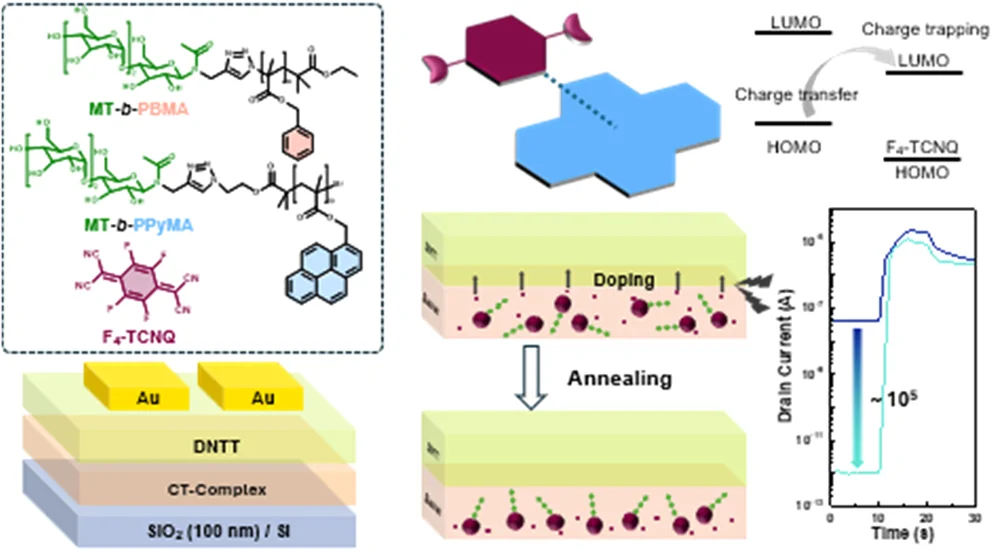

## Introduction

The rapid development of artificial intelligence, machine learning, and big data analytics has created unprecedented demands for data storage and processing. Conventional von Neumann architectures struggle to meet these demands because processing and memory units are physically separated. The frequent transfer of data between these units has therefore become a major performance bottleneck.^1^ To address this “memory wall,” considerable effort has been devoted to developing nonvolatile memory (NVM) devices^2–4^ with high storage density, low power consumption, and excellent scalability. Among emerging technologies, organic field-effect transistor (OFET)-based memory devices have attracted considerable attention as a versatile platform for next-generation electronics.^5–9^ In particular, integrating photosensitivity into OFET memory architectures enables light sensing and data storage within a single device, providing a promising route toward advanced optoelectronic systems.^10–13^ Moreover, these phototransistor memory devices can be programmed using both optical and electrical stimuli, allowing multiple distinguishable logic states to be encoded in a single device. Such multilevel storage capability substantially increases information density without enlarging the physical device footprint.^14–16^

However, achieving precise and stable control over photoinduced charge transport and trapping remains a major challenge in organic electronics. The generation, migration, and storage of photogenerated carriers are strongly influenced by the energetic landscape, interfacial quality, and molecular organization of the active materials. Uncontrolled trap distributions and morphological instability can promote charge leakage, cause variations in programming behavior, and deteriorate data retention. To address these issues, various charge-storage mechanisms and functional materials have been explored, including floating gates,^17–20^ ferroelectric materials,^21–24^ small-molecule charge-trapping layers,^25–28^ and organic electrets.^29–33^ Among these approaches, organic electrets are particularly promising because their chemical structures, energy levels, and charge-trapping characteristics can be readily tailored through molecular design. Their excellent solution processability and compatibility with organic semiconductors also facilitate the fabrication of structurally diverse memory devices. Moreover, supramolecular engineering provides an effective strategy for regulating charge storage at the molecular level. By exploiting noncovalent interactions such as hydrogen bonding,^34,35^ host–guest interactions,^36^ and the formation of charge-transfer (CT) complexes,^37,38^ the molecular arrangement and local electronic environment of the electret layer can be systematically modified.

Among these supramolecular approaches, donor–acceptor CT electrets have emerged as an effective molecular strategy for enhancing charge trapping and improving memory performance in OFET-based memory devices. By introducing electron-accepting chromophores into polymer electrets through intermolecular CT interactions, well-defined trapping sites can be generated, leading to enlarged memory windows and improved charge retention. For example, supramolecular electrets based on donor–acceptor charge-transfer complexes have been demonstrated to effectively enhance charge trapping and memory characteristics in transistor memory devices.^37,39^ In particular, F_4_-TCNQ has attracted increasing attention as a high-electron-affinity molecular acceptor because of its efficient donor–acceptor charge-transfer capability and has been employed to construct charge-transfer systems and charge-trapping layers for organic transistor memories and related optoelectronic devices.^40^ These examples highlight the potential of donor–acceptor molecular engineering for constructing high-performance organic memory devices while providing a versatile platform for tuning charge-storage behavior.

Despite these advantages, incorporating functional guest molecules or dopants into organic materials often creates a trade-off between enhanced charge storage and the preservation of the host matrix’s structural integrity. Therefore, chemical doping strategies continue to face significant challenges because dopant incorporation can introduce undesirable structural and electrical effects.^41,42^ As the dopant concentration increases, maintaining a uniform molecular distribution within the host matrix becomes increasingly difficult. Specifically, dopant molecules may disrupt the molecular packing of the host, leading to increased structural disorder and reduced charge-carrier mobility.^43–45^ Furthermore, poor molecular compatibility between the host and guest components further promotes dopant aggregation and phase separation, thereby compromising morphological stability and operational reliability. The uncontrolled spatial distribution of dopants may also produce nonuniform trapping sites or localized conductive pathways, ultimately resulting in excessive leakage current and a reduced memory ratio.^46^ These effects ultimately hinder reliable multilevel operation, highlighting the importance of controlling both the molecular interactions and spatial organization of dopants within organic memory materials. To mitigate these challenges, one effective strategy is to introduce sterically bulky substituents or conjugation-interrupted insulating spacers that spatially confine charge carriers and suppress parasitic leakage pathways.^47,48^

In this work, we demonstrate high-performance organic phototransistor memory devices based on a series of carbohydrate-based block copolymer (BCPs) as multifunctional electrets. Two distinct BCP materials, maltotriose-*block*-poly­(1-pyrenemethyl methacrylate) (PPy) and maltotriose-*block*-poly­(benzyl methacrylate) (PB), are investigated, featuring different pendant π-electron donor groups. Incorporating 2,3,5,6-tetrafluoro-7,7,8,8-tetracyanoquinodimethane (F_4_-TCNQ) into these BCP matrices, supramolecular CT complexes are formed. Within these device architectures, the CT complexes serve as primary charge-trapping centers, where donor–acceptor interactions generate CT-assisted trapping sites, enabling stable multilevel storage. With annealing treatment, the BCP electrets undergo molecular reorganization, accompanied by a redistribution of F_4_-TCNQ within the electret layer. This redistribution enables both PPy and PB systems to accommodate substantially higher concentrations of F_4_-TCNQ while maintaining stable device operation. Rather than generating additional trapping sites, the annealing treatment optimizes the spatial distribution of F_4_-TCNQ within the BCP matrices. It effectively mitigates structural perturbations and unfavorable charge-leakage pathways induced by excessive dopant incorporation, thereby preserving the integrity of the supramolecular framework and maintaining stable memory characteristics and reliable current output. The synergistic integration of the carbohydrate-based BCP framework, donor–acceptor CT interactions, and annealing-induced molecular reorganization provides an effective strategy for realizing stable, high-performance organic optoelectronic memory devices.

## Results and Discussion

### Design of the Supramolecular Electret Systems

To achieve stable multilevel data storage and overcome the phase-separation and leakage issues prevalent in conventional doped systems, a series of functionally designed block copolymer electrets was rationally designed. Figure 1a illustrates the architecture of the phototransistor memory device and the corresponding charge-transfer (CT) supramolecular electret systems. In this molecular design, the maltotriose carbohydrate block provides a robust insulating domain to stabilize the supramolecular framework, while the pendant aromatic groups in the PBMA and PPyMA segments act as π-electron donors. To construct the supramolecular electret materials, poly­(benzyl methacrylate) (PBMA) and poly­(1-pyrenemethyl methacrylate) (PPyMA) were first synthesized via atom transfer radical polymerization (ATRP) using functional initiators. Subsequently, the resulting azide-functionalized polymers were prepared and further reacted with alkyne-functionalized maltotriose via CuBr-catalyzed click chemistry to yield maltotriose-*block*-poly­(benzyl methacrylate) (PB) and maltotriose-*block*-poly­(1-pyrenemethyl methacrylate) (PPy). The detailed synthetic methods are summarized in the Supporting Information, and their chemical structure characterizations are presented in Figures S1–S4 (Supporting Information). To fabricate the supramolecular electret layers, PB and PPy were blended with different concentrations of F_4_-TCNQ to construct the CT supramolecular electret. The electret systems were assigned as T5 (5 mol %), T10 (10 mol %), and T25 (25 mol %) according to the composition ratio, as denoted in Table S1 (Supporting Information), where the numbers in parentheses represent the molar ratios of F_4_-TCNQ to the repeating unit of the polymers (benzyl group in PB and pyrene group in PPy). The thin-film electrets were fabricated by spin coating and subsequently subjected to sequential solvent-vapor annealing and thermal annealing at 140 °C to optimize molecular organization and dopant distribution within the block copolymer, thereby enhancing dopant accommodation. After the annealing process, dinaphtho­[2,3-b:2′,3′-*f*]­thieno­[3,2-*b*]­thiophene (DNTT) and Au electrodes were thermally evaporated as the organic semiconductor channel layer and source/drain electrodes, respectively, to complete the bottom-gate/top-contact phototransistor memory devices.

**Figure 1:**
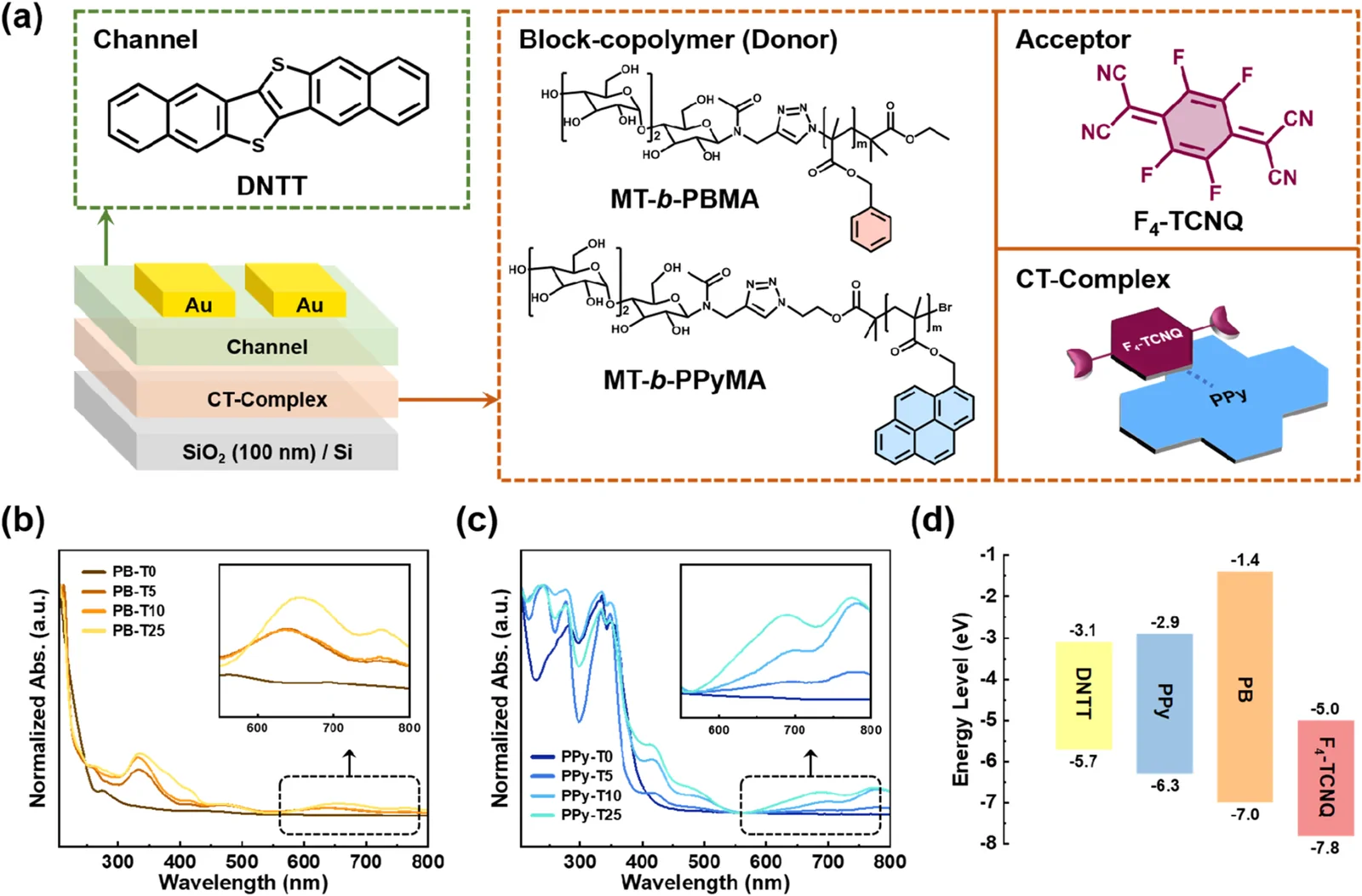
(a) Schematic illustration of the phototransistor memory device and the chemical structures of the constituent materials in the electret and active layers. UV–vis absorption spectra of the (b) PB-based and (c) PPy-based supramolecular films. (d) Energy level diagrams of DNTT, PPy, PB, and F_4_-TCNQ.

### Optical Characterizations and CT Mechanisms

To investigate the photoinduced intermolecular CT interactions within the supramolecular electret systems, UV–vis absorption spectroscopy was first performed, and the corresponding spectra are presented in Figure 1b,c. After incorporating F_4_-TCNQ, both PB- and PPy-based films exhibited additional absorption features. Specifically, the PPy systems showed enhanced absorption in the 400–450 nm region, whereas the PB systems displayed more pronounced spectral changes spanning approximately 300–500 nm. Comparison with the absorption spectrum of pristine F_4_-TCNQ (Figure S9, Supporting Information) shows that these absorption features partially overlap with the intrinsic absorption of F_4_-TCNQ. Therefore, the spectral changes observed in the 300–500 nm region likely arise from both the intrinsic absorption of F4-TCNQ and donor–acceptor interactions, making it difficult to clearly distinguish their individual contributions from UV–vis spectra alone. Similar spectral overlap has also been reported for F_4_-TCNQ-based donor–acceptor systems.^49,50^ Notably, a broad absorption feature extending from approximately 600 to 800 nm was observed only in the PB/F_4_-TCNQ and PPy/F_4_-TCNQ films, whereas no corresponding absorption was detected for pristine F_4_-TCNQ. Consistent with previous studies identifying the low-energy absorption as a characteristic of F_4_-TCNQ radical anions,^51^ the newly observed spectral features strongly support the formation of CT complexes between the donor blocks and F_4_-TCNQ. Nevertheless, because absorption may arise from contributions from different charged species and intermolecular electronic transitions, UV–vis spectra alone are insufficient to definitively determine the exact CT state. Accordingly, the intermolecular CT interactions were further examined using energy-level analysis, photoluminescence (PL), and time-resolved photoluminescence (TRPL) measurements.

To further understand the intermolecular CT interactions, the materials’ energy levels were analyzed. The optical bandgap was determined from the UV–vis absorption onset using Tauc plots, while the highest occupied molecular orbital (HOMO) levels of the BCPs were estimated from cyclic voltammetry (CV) measurements (Figures S10 and S11, Supporting Information). The corresponding lowest unoccupied molecular orbital (LUMO) levels were subsequently calculated from the HOMO energy and optical bandgap, and the resulting energy-level diagram is depicted in Figure 1d. Compared with PB (HOMO = −7.0 eV), PPy exhibited a higher HOMO level of −6.3 eV, resulting in a more favorable energetic alignment with the LUMO of F_4_-TCNQ (−5.0 eV). Such an energetic configuration is expected to facilitate photoinduced intermolecular CT and charge separation. In contrast, the larger energy offset between PB and F_4_-TCNQ may limit the efficiency of donor–acceptor interactions and subsequent CT processes.

Furthermore, PL spectroscopy was performed under an excitation wavelength of 375 nm to evaluate the excited-state CT behavior. Only the PPy systems were further analyzed because the PB-based films exhibited negligible fluorescence emission. As shown in Figure 2a, pristine PPy films exhibited strong fluorescence emission originating from the pyrene moieties. After incorporating F_4_-TCNQ, significant fluorescence quenching was observed, indicating efficient photoinduced donor–acceptor interactions accompanied by intermolecular charge transfer. Such fluorescence quenching is widely reported as a characteristic signature of photoinduced charge transfer in donor–acceptor systems, in which charge separation introduces efficient nonradiative recombination pathways that suppress fluorescence emission.^52,53^ The PL intensity decreased progressively as the F_4_-TCNQ concentration increased, indicating enhanced nonradiative CT pathways at higher F_4_-TCNQ loadings. In particular, the PL emission integrals decreased by approximately 82% for PPy-T10 and reached a maximum quenching efficiency of 96% for PPy-T25 compared with pristine PPy. The detailed PL intensity variations and quenching efficiencies are summarized in Table S2 (Supporting Information). Interestingly, after annealing, the fluorescence quenching was partially suppressed, resulting in a noticeable recovery of PL intensity in the annealed PPy samples. In the as-cast PPy-T25 films, the stronger PL quenching indicates more extensive donor–acceptor interactions between PPy and F_4_-TCNQ. However, the increased polymer chain mobility during annealing enables molecular reorganization and dopant redistribution within the supramolecular framework. This structural refinement effectively moderates the excessive nonradiative charge-transfer pathways present in the as-cast state. As a result, the PL intensity partially recovers while retaining the effective CT-assisted interactions necessary for stable memory operation.

**Figure 2:**
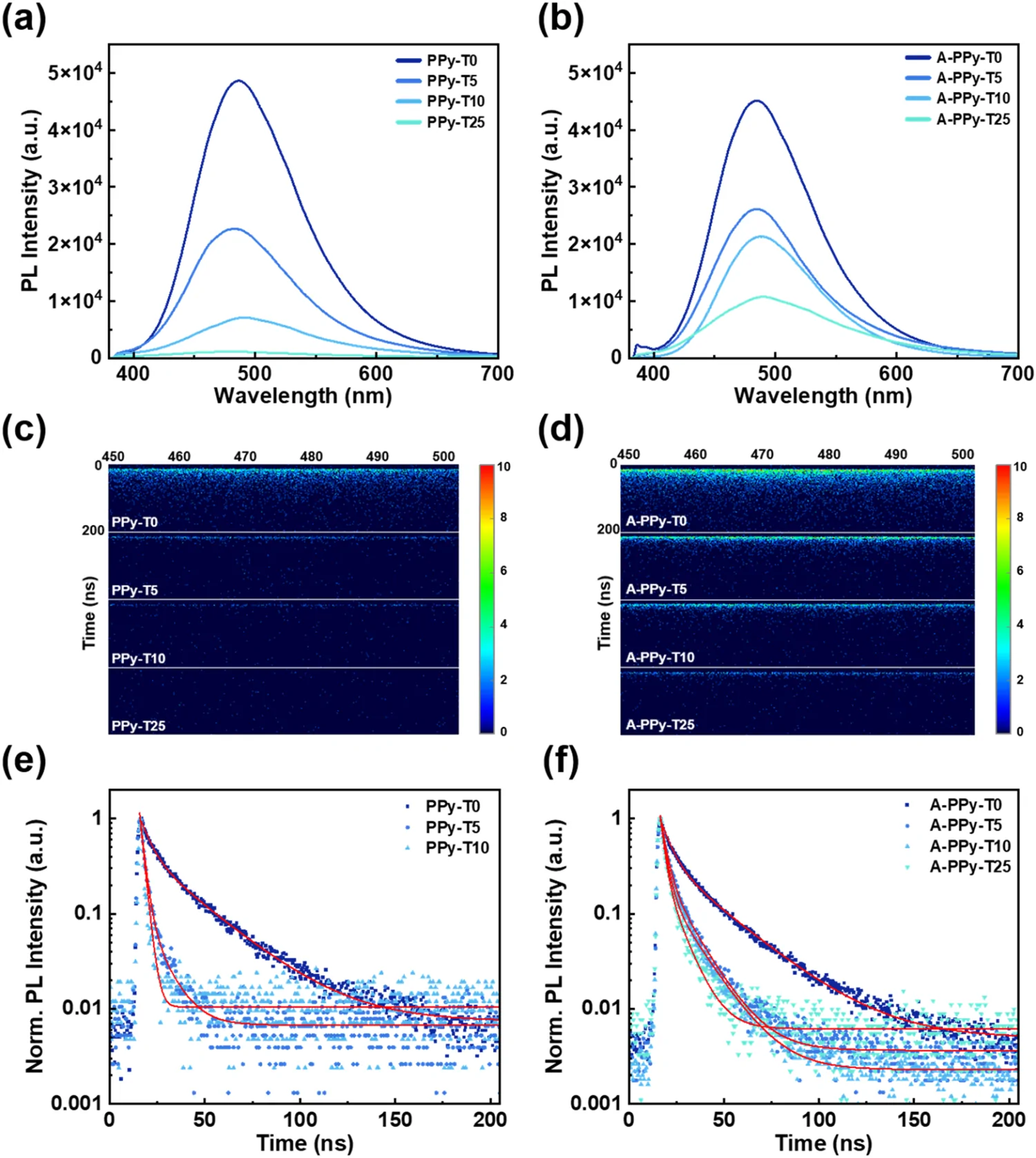
(a, b) Steady-state PL emission spectra of the supramolecular films with an excitation wavelength of 375 nm. (c, d) 2D TRPL patterns and (e, f) 1D decay profiles: (a, c, e) PPy systems and (b, d, f) annealed PPy systems.

The excited-state carrier dynamics were further investigated by TRPL measurements (Figure 2c–f). The PL decay profiles were analyzed using biexponential fitting to obtain the short-lived lifetime (τ_1_), long-lived lifetime (τ_2_), and intensity-averaged lifetime (τ_avg_), as summarized in Table S3 (Supporting Information). Compared with pristine PPy films, the F_4_-TCNQ-doped samples exhibited substantially shortened fluorescence lifetimes, indicating that intermolecular CT produces efficient nonradiative decay pathways. The average fluorescence lifetime decreased from 9.39 ns for pristine PPy to 1.93 ns for PPy-T10, further confirming the accelerated excited-state CT process after F_4_-TCNQ incorporation. Moreover, as the F_4_-TCNQ concentration increases, the fluorescence decays progressively faster. For the PPy-T25 sample, the fluorescence signal became too weak to obtain reliable fitting parameters because of severe fluorescence quenching at high F_4_-TCNQ loading, further supporting enhanced excited-state intermolecular CT. After annealing, the fluorescence lifetimes increased relative to the corresponding as-cast samples, consistent with the recovered PL intensity. This result suggests that annealing treatment modifies the donor–acceptor interactions while maintaining efficient photoinduced charge transfer. Collectively, the optical characterizations provide compelling and complementary evidence for the formation of donor–acceptor CT complexes within the PPy/F4-TCNQ supramolecular electrets. Crucially, these CT interactions can be precisely modulated by both the dopant concentration and the annealing-induced molecular reorganization, which ultimately dictate the subsequent photomemory characteristics of the devices.

### Electrical Performance and Memory Behavior of the Phototransistor Memory

To investigate the influence of CT characteristics on memory behavior, supramolecular electret systems were integrated into phototransistor memory devices for electrical characterization. The transfer characteristics of the devices are presented in Figures 3a–d and S12 (Supporting Information). All measurements were conducted under a drain voltage (*V*_d_) of −60 V with the gate voltage (*V*_g_) swept from 20 to −60 V. The photowriting (PW) process was performed by illuminating the devices with 365 nm light (1.44 mW cm^–2^) for 10 s under *V*_d_ = −60 V and *V*_g_ = 0 V, while electrical erasing (EE) was achieved by applying *V*_g_ = −60 V and *V*_d_ = 0 V for 1 s. In the PB-based systems, the pristine PB-T0 device exhibited only a limited memory window because the PB matrix itself possesses relatively weak intrinsic charge-trapping capability. After incorporating F_4_-TCNQ, the transfer curves gradually shifted toward negative potentials, accompanied by enlarged threshold voltage shifts (Δ*V*_th_), indicating enhanced charge trapping within the electret layer (Figures 3a and S12a,c, Supporting Information). The Δ*V*_th_ increased from 5.3 V for PB-T0 to 31.7 V for PB-T10, where the maximum memory window was obtained. However, when the F_4_-TCNQ concentration was further increased to PB-T25, the Δ*V*_th_ decreased to 19.1 V, indicating that 25 mol % F_4_-TCNQ adversely affects the effective charge-trapping behavior (Figure 3c). The PB matrix itself exhibited negligible memory characteristics and relatively weak donor properties due to the large energy-level mismatch between the benzyl side groups and F_4_-TCNQ. Therefore, the observed Δ*V*_th_ shift does not result from efficient intermolecular CT interactions, but rather from the strong electron-withdrawing ability of F_4_-TCNQ, which modifies the charge distribution within the device and induces a negative shift in the transfer characteristics. Moreover, the Δ*V*_th_ significantly decreased when the F_4_-TCNQ loading was further increased to 25 mol %. This behavior suggests that excessive F_4_-TCNQ increases the electrical influence of the electret layer on the DNTT channel, possibly through unintended p-doping and leakage pathways. Interestingly, after annealing, the Δ*V*_th_ partially recovered in high-dopant-concentration systems, suggesting that annealing mitigates the adverse effects of excessive F_4_-TCNQ incorporation and modifies its organization within the electret layer. In contrast, the PPy-based devices exhibited intrinsically large memory windows even in the absence of F_4_-TCNQ because the pyrene-containing BCP itself functions as an efficient electret material (Figure 3b). After introducing F_4_-TCNQ, the Δ*V*_th_ further increased from 50.4 V for PPy-T0 to 56.7 V for PPy-T10 (Figures 3b and S12e,g, Supporting Information), indicating optimized CT-assisted charge trapping at this F_4_-TCNQ loading. Consistent with the PL and TRPL results discussed earlier, the favorable energetic alignment between the pyrene donor groups and F_4_-TCNQ acceptors facilitates efficient photoinduced intermolecular CT interactions, thereby generating additional charge-trapping states and enhancing memory performance. Similar to the PB systems, further increasing the F_4_-TCNQ loading to 25 mol % decreased the Δ*V*_th_ of PPy-T25 to 35.9 V (Figure 3d). The reduced memory window suggests that excessive F_4_-TCNQ incorporation and highly disordered donor–acceptor interactions begin to interfere with efficient charge trapping, thereby compromising device stability and memory performance. Nevertheless, compared with PB systems, the annealing treatment produced relatively minor changes in the Δ*V*_th_ of PPy-based devices, suggesting that the stronger intrinsic charge-trapping capability of the pyrene-containing electret play a dominant role in determining the memory window.

**Figure 3:**
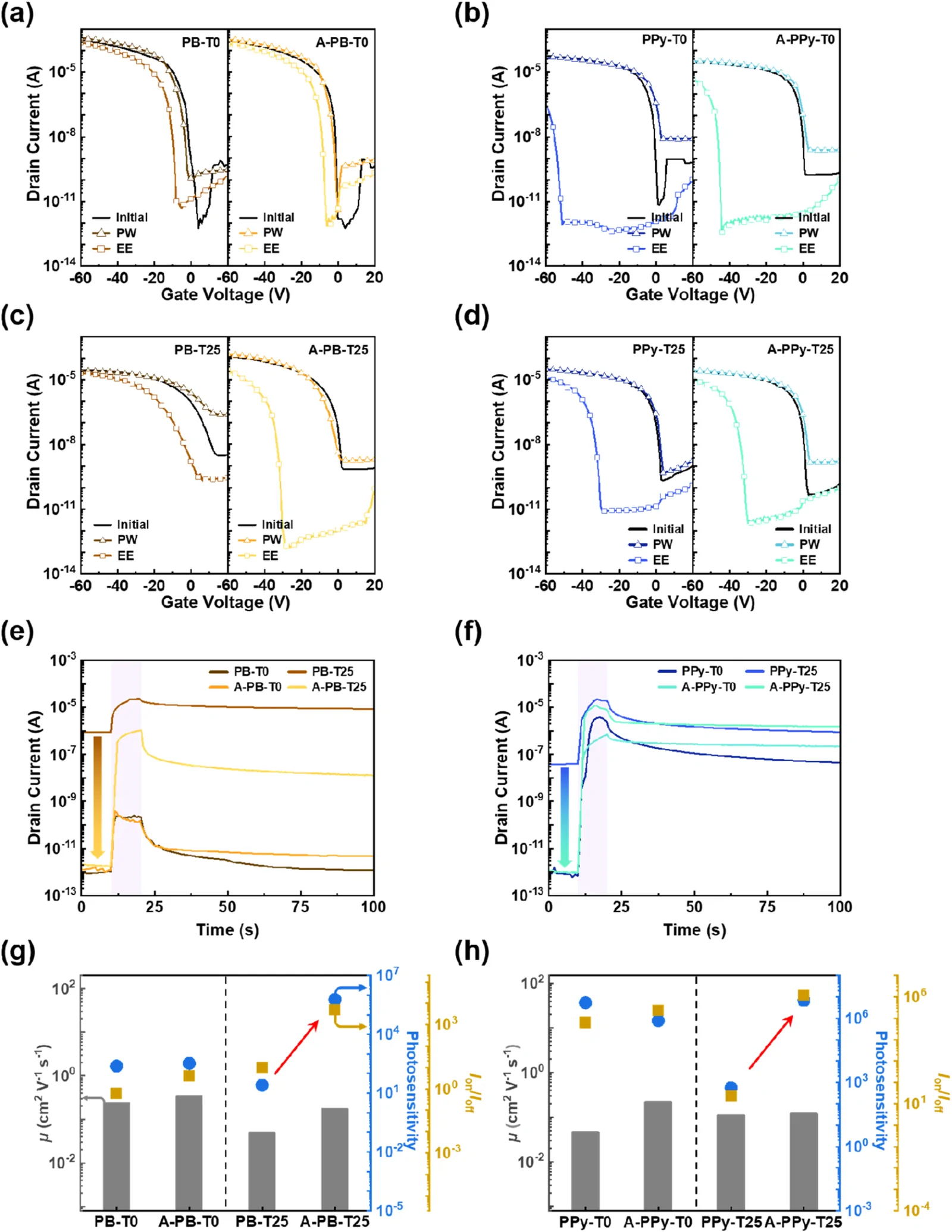
(a–d) Transfer characteristics, (e, f) transient characteristics, and (g, h) summary of the memory device parameters: (a) PB-T0 and A-PB-T0, (b) PPy-T0 and A-PPy-T0, (c) PB-T25 and A-PB-T25, (d) PPy-T25 and A-PPy-T25, (e, g) PB-based supramolecular electret, and (f, h) PPy-based supramolecular electret. Note that the transfer curves were measured under *V*_d_ = −60 V and *V*_g_ from 20 to −60 V, and the photowriting (PW) was performed under 365 nm light illumination for 10 s at *V*_d_ = −60 V, whereas electrical erasing (EE) was conducted by applying *V*_g_ = −60 V for 1 s.

To quantitatively evaluate the effective charge-trapping capability associated with the observed threshold-voltage shifts, capacitance–voltage (C–V) measurements were performed, and the corresponding C–V characteristics are presented in Figure S13 (Supporting Information) to extract electret capacitances and the calculated trap densities, which are summarized in Table S4 (Supporting Information). The electret capacitances exhibited only minor variations among the different samples (22.5–24.5 nF cm^–2^), indicating that the calculated trap-density differences are primarily governed by the corresponding threshold-voltage shifts. The trap-density trend closely follows the evolution of the memory window, increasing at moderate F_4_-TCNQ loading and decreasing at 25 mol % F_4_-TCNQ for both the PB- and PPy-based systems. These results provide quantitative electrical evidence that appropriate donor–acceptor interactions promote effective charge trapping, whereas excessive F_4_-TCNQ incorporation no longer contributes to additional effective trapping sites. Notably, the PPy-T25 and A-PPy-T25 devices exhibited comparable trap densities, indicating that the annealing treatment does not substantially alter the density of effective trapping sites.

To evaluate the charge-trapping behavior associated with the observed threshold-voltage shifts, dual-sweep transfer measurements were performed, as shown in Figure S14 (Supporting Information). As shown in Figures S14a,b, both the pristine and F_4_-TCNQ -containing PPy devices exhibited clear hysteresis between the forward and backward gate-voltage sweeps before and after annealing. The observed hysteresis is consistent with charge trapping within the supramolecular electret layer and further supports that the threshold-voltage shifts originate from memory-related trapping processes. To ensure the reliability of these findings, three independently fabricated optimized A-PPy-T25 devices were evaluated. As presented in Figure S14c, five dual-sweep measurements collected from these devices show highly consistent transfer characteristics and hysteresis window. In addition, this hysteresis was robustly maintained across varying gate-voltage sweep rates (1, 0.5, and 0.1 V s^–1^, Figure S14d). Collectively, these results confirm that the memory behavior stems from intrinsic charge trapping within the supramolecular electret, effectively ruling out transient measurement artifacts and demonstrating excellent reproducibility. To further elucidate the charge-trapping behavior and photoresponse characteristics of the supramolecular electret systems, transient photocurrent measurements were performed, and the corresponding results are shown in Figures 3e,f and S15 (Supporting Information). In the PB-based devices, the pristine PB-T0 system exhibited only a limited photocurrent response and relatively poor retention, consistent with its weak intrinsic charge-trapping capability. After incorporating F_4_-TCNQ, the photocurrent response progressively increased with increasing F_4_-TCNQ loading, consistent with the increased trap density observed at moderate dopant concentrations. However, further increasing the F_4_-TCNQ loading, particularly in the PB-T25 device, resulted in unstable current output and a substantially elevated off-state current reaching 39 nA. This behavior suggests that the highly loaded F_4_-TCNQ introduces undesirable leakage pathways and increased interference with charge transport in the DNTT channel layer. Following the annealing treatments, the transient characteristics became significantly more stable, accompanied by a pronounced reduction in leakage current. Consequently, the photosensitivity and *I*_on_/*I*_off_ ratio increased by approximately two and 4 orders of magnitude, respectively (Figure 3g). These improvements are more likely associated with annealing-induced reorganization of the electret layer and reduced leakage conduction rather than an increase in the strength of the intermolecular CT interactions. Considering the relatively weak donor characteristics of the PB system and the partial recovery of PL intensity after annealing, the annealing treatment likely reorganizes the supramolecular electret, thereby reducing the electrical influence of excessive F_4_-TCNQ on the DNTT channel while preserving the effective charge-trapping capability of the electret. In contrast, the PPy-based systems exhibited stronger and more stable photoresponses because the pyrene-containing BCP possesses superior intrinsic charge-trapping capability, as shown in Tables 1 and S5 (Supporting Information). Notably, the pristine PPy-T0 device exhibited a stepwise increase in photocurrent under illumination, whereas this stepwise/discontinuous response became less pronounced after incorporating F_4_-TCNQ, resulting in more rapid photocurrent accumulation. This result indicates that the intermolecular CT interactions between the pyrene groups (donor) and F_4_-TCNQ (acceptor) facilitate more efficient charge separation and trapping processes within the electret layer. Among the as-cast devices, PPy-T10 exhibited the most balanced electrical performance, combining the largest memory window and trap density with high photosensitivity, a high *I*_on_/*I*_off_ ratio, and stable transient characteristics. However, when the F_4_-TCNQ concentration was further increased to 25 mol % (PPy-T25), the trap density decreased, while the transient characteristics deteriorated and the leakage current increased markedly, leading to substantial reductions in both the *I*_on_/*I*_off_ ratio and photosensitivity (Figure 3h). To further verify the critical role of the carbohydrate block in suppressing leakage conduction, control experiments were performed with the reference homopolymers PPyMA and PBMA, which lack the carbohydrate block. These homopolymers were blended with F_4_-TCNQ and served as CT supramolecular electrets in photomemory. As shown in Figure 4a,b, although annealing treatments were also applied to the homopolymer systems, the off-state current exhibited only limited reduction compared with the carbohydrate-containing BCP systems. This result demonstrates that annealing treatment alone is insufficient to achieve enhanced device stability. Instead, the carbohydrate block plays an essential role in enabling the annealing-induced molecular reorganization responsible for the improved electrical characteristics.

**Figure 4:**
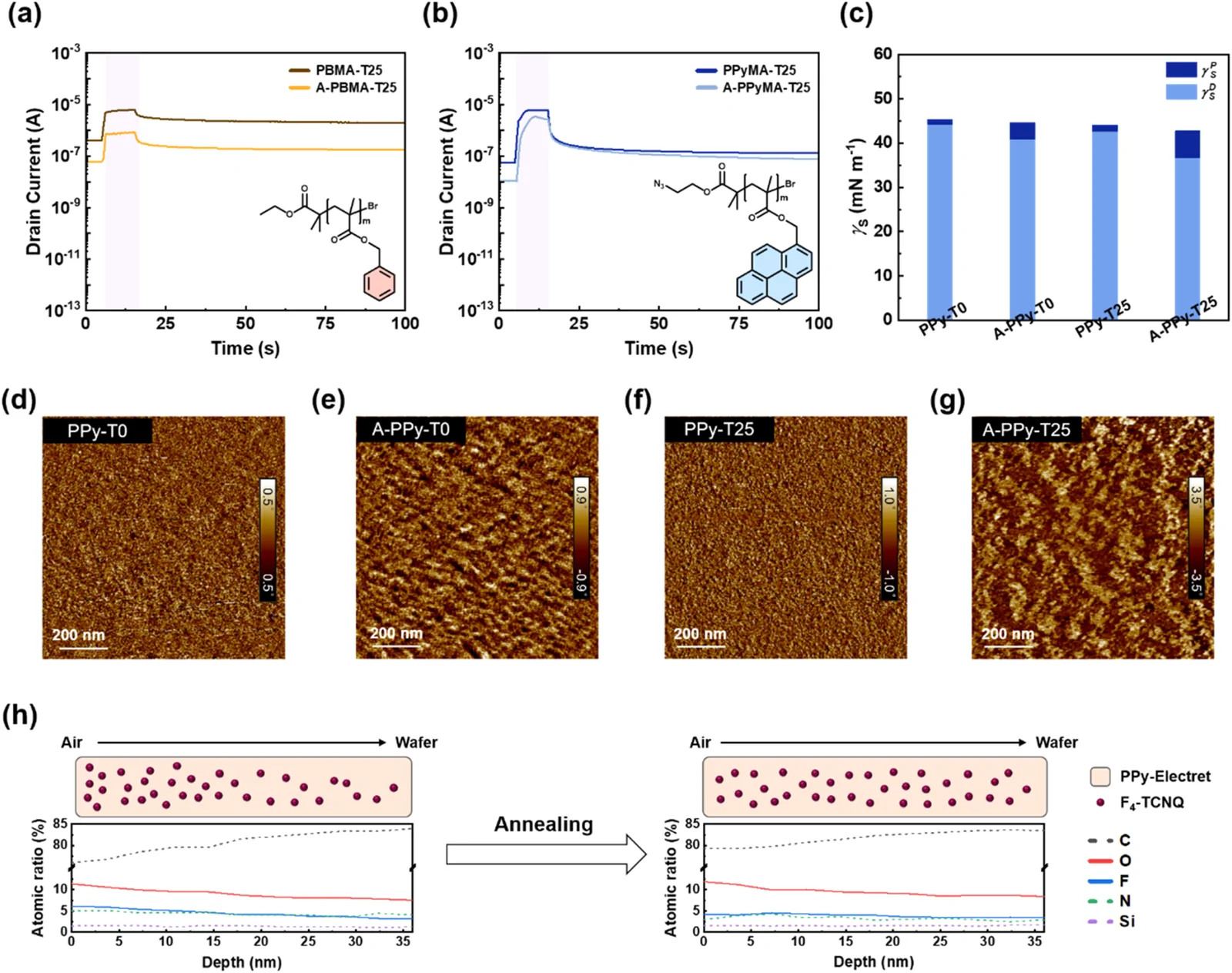
(a) Surface free energies and their polar and dispersion fraction of PB- and PPy-based electret films before and after annealing. Transient characteristics of devices comprising reference homopolymers: (b) PBMA/F_4_-TCNQ and (c) PPyMA/F_4_-TCNQ, without the carbohydrate block, before and after annealing treatment. AFM phase images of (d) PPy-T0, (e) A-PPy-T0, (f) PPy-T25, and (g) A-PPy-T25. (h) Schematic illustration of the annealing-induced reorganization of the carbohydrate-based CT supramolecular electret, together with the corresponding XPS depth profiles before and after annealing.

**Table 1:** Device Characteristics of Organic Phototransistor Memory with the Carbohydrate-Based BCP CT Supramolecular Electrets and DNTT as a Semiconducting Channel

electret	μ [cm^2^ V^–1^ s^–1^]^a^	*ΔV*_th_ [V]^b^	*I*_on_ / *I*_off_ ^c^	photosensitivity^d^	responsivity [mA W^–1^]^e^
PB-T0	0.24	5.3	6.20 × 10^1^	2.41 × 10^2^	3.54 × 10^–5^
A-PB-T0	0.35	4.7	4.10 × 10^0^	3.32 × 10^2^	5.63 × 10^–5^
PB-T25	0.05	19.1	1.01 × 10^1^	2.58 × 10^1^	32.0
A-PB-T25	0.17	29.0	7.35 × 10^3^	5.91 × 10^5^	1.5
PPy-T0	0.25	50.4	6.32 × 10^4^	5.32 × 10^6^	5.1
A-PPy-T0	0.22	47.2	2.40 × 10^5^	7.78 × 10^5^	1.0
PPy-T25	0.11	35.9	2.26 × 10^1^	5.57 × 10^2^	30.6
A-PPy-T25	0.12	36.7	1.61 × 10^6^	1.27 × 10^7^	9.1

a Mobility obtained from the saturation regime of the initial transfer characteristics. Power of light sources: 365 nm, 1.44 mW cm^–2^.

b Memory window: Δ*V*_th_ = *V*_th,write_ – *V*_th,erase_.

c The ratio of photocurrent to dark current.

d Photosensitivity: *P* = (*I*_on_ - *I*_off_)/*I*_off_.

e Responsivity: *R* = (*I*_on_ – *I*_off_)/(*P*_opt_ × A), *P*_opt_ is the incident optical power density (1.44 mW cm^–2^), and *A* is the effective channel area.

The postillumination charge-retention behavior was further evaluated using the retention ratio extracted from the transient photocurrent measurements, as summarized in Table S5 (Supporting Information). Notably, the annealed PPy-based devices consistently exhibited higher retention ratios than their corresponding as-cast devices. For example, the memory current retention in Figure 3e,f, defined as the drain current ratio at 80 and 1 s after photowriting, increased from 13.0% for PPy-T10 to 41.8% for A-PPy-T10 and from 6.2% for PPy-T25 to 24.7% for A-PPy-T25. These results indicate that annealing improves the persistence of the programmed current while maintaining comparable trap densities. Remarkably, the performance deterioration observed in the PPy-T25 device was effectively alleviated after annealing. Upon annealing treatment, the off-state leakage current of the PPy-T25 device decreased dramatically from 39 nA to 0.95 pA, corresponding to a reduction of approximately 5 orders of magnitude. As a result of the substantially suppressed background current, the annealed PPy-T25 device (A-PPy-T25) significantly outperformed its as-cast counterpart and even exceeded the performance of the optimized as-cast PPy-T10 device, delivering an exceptional *I*_on_/*I*_off_ ratio of 1.61 × 10^6^ and a high photosensitivity of 1.27 × 10^7^. Combined with the trap density and retention ratio, these results indicate that the annealing-induced supramolecular reorganization does not generate additional trapping sites but instead improves the utilization of the existing CT-assisted trapping pathways by reducing the electrical influence of excessive F_4_-TCNQ on the DNTT channel. Benchmark comparison with representative organic phototransistor memory (Table S6, Supporting Information) indicates that the present device delivers competitive electrical performance rather than record-high metrics. The A-PPy-T25 device achieves an *I*_on_/*I*_off_ ratio of 1.61 × 10^6^ under a relatively mild photowriting condition (365 nm, 1.44 mW cm^–2^, 10 s), highlighting the effectiveness of the carbohydrate-based CT supramolecular electret design. Consistent with the optical characterizations, the partial recovery of PL intensity after annealing suggests that the donor–acceptor interactions are reorganized rather than eliminated, while efficient CT-assisted trapping sites remain available for stable photomemory operation.

### Interfacial Reorganization and Mechanism of the Annealing-Enhanced Device Stability

To further clarify the origin of the annealing-enhanced device performance, the surface morphology of the supramolecular electret films was systematically investigated using atomic force microscopy (AFM), as shown in Figures 4d–g, and S16 and S17 (Supporting Information). The surface roughness (*R*_q_) showed minimal variation after annealing, indicating that the enhanced device performance cannot be simply attributed to improved film smoothness or reduced surface roughness. In addition, no obvious aggregates or large grains were observed in either the AFM height or phase images after F_4_-TCNQ incorporation, suggesting that macroscopic aggregation or crystallization of F_4_-TCNQ did not occur under the present processing conditions. Nevertheless, although the height images remained relatively unchanged, noticeable differences were observed in the AFM phase images after annealing, indicating that the annealing treatment influences the molecular organization of the electret. These observations suggest that the improved device performance originates from annealing-induced molecular reorganization rather than changes in surface roughness alone.

To observe the interfacial changes associated with this structural reorganization, surface-energy analysis was conducted through contact-angle measurements. As shown in Figure 4c, the polar component (γ^P^ S) of the surface energy increased for both the PB- and PPy-based electret films after annealing. Specifically, the γ^P^ S of the PPy-T25 film significantly increased from 1.5 to 6.1 mN m^–1^ after annealing, indicating a substantial enhancement in surface polarity. Considering that the maltotriose block represents the primary polar component in the carbohydrate-based block copolymer, the increased surface polarity is likely associated with preferential enrichment of the carbohydrate segments near the film surface after annealing treatment. Such molecular reorganization suggests that annealing alters the molecular organization of the carbohydrate-based block copolymer, which is expected to influence the depth profile of F_4_-TCNQ within the electret layer.

To directly examine whether annealing modifies the distribution of F_4_-TCNQ, XPS depth profiling was performed, as shown in Figure 4h. Before annealing (PPy-T25), the fluorine signal was strongest at the film surface and gradually decreased with increasing etching depth, indicating preferential enrichment of F_4_-TCNQ near the surface. After annealing (A-PPy-T25), the fluorine signal became substantially more uniform throughout the film thickness, demonstrating that annealing modifies the depth profile of F_4_-TCNQ within the electret layer. Combined with the homopolymer control experiments discussed above, these results suggest that the carbohydrate block enables the annealing-induced reorganization responsible for the modified F_4_-TCNQ depth profile. This interpretation is further supported by the optical characterizations discussed above. Following annealing treatment, the partial recovery of PL intensity, together with the prolonged fluorescence lifetime, indicates that donor–acceptor interactions are more effectively regulated. These optical results are consistent with the modified F_4_-TCNQ depth profile revealed by the XPS analysis, suggesting that annealing improves the utilization and retention of trapped charges while suppressing leakage current. Based on these observations, a schematic mechanism is proposed in Figure 4h. In the as-cast state, the XPS depth profile indicates that F_4_-TCNQ is preferentially enriched near the surface of the electret layer, resulting in severe fluorescence quenching, increased interaction with the DNTT channel, and unstable electrical characteristics. After annealing, molecular reorganization of the BCP modifies the depth profile of F_4_-TCNQ, resulting in a more homogeneous vertical distribution throughout the electret layer. This modified depth profile is consistent with reduced interaction between F_4_-TCNQ and the DNTT channel while maintaining effective donor–acceptor charge-transfer interactions for charge trapping. Consequently, the annealing-induced molecular reorganization enabled by the carbohydrate block is associated with suppressed leakage current, improved charge retention, and enhanced photomemory performance and operational stability.

To further evaluate the generality of the proposed strategy, tris­(pentafluorophenyl)­borane (BCF), a representative Lewis-acid molecular dopant widely used for p-type doping of organic semiconductors, was selected as an alternative dopant.^54^ BCF has been demonstrated to effectively dope a wide range of semiconducting polymers, making it an appropriate model system for examining whether the proposed annealing strategy can be extended beyond the F_4_-TCNQ system.^54^ The corresponding electrical characteristics are presented in Figure 5. Similar to the F_4_-TCNQ-based devices, the BCF-doped devices exhibited a pronounced reduction in the off-state leakage current after annealing while maintaining stable photomemory characteristics. Consequently, the annealed BCF devices exhibited a significantly improved *I*_on_/*I*_off_ compared with their as-cast counterparts. These results indicate that the beneficial effect of the carbohydrate-based BCP is not limited to the integer-charge-transfer dopant system (F_4_-TCNQ). Instead, the annealing-induced molecular reorganization of the carbohydrate-based BCP effectively suppresses leakage current while preserving efficient charge-trapping behavior in another representative molecular dopant. Furthermore, BCF has also been successfully employed in organic phototransistors,^55^ suggesting that the proposed carbohydrate-based block copolymer strategy is potentially applicable to a broader class of molecularly doped organic phototransistor memory devices.

**Figure 5:**
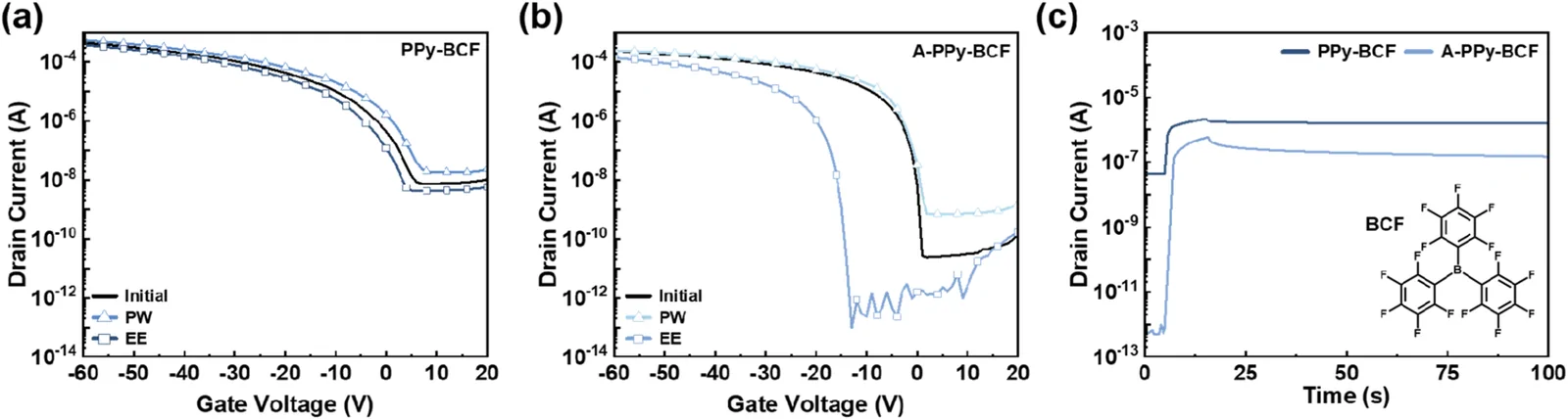
Reference phototransistor memory device comprising the PPy BCP electret and a Lewis acid dopant of BCF in comparison to the integer-charge-transfer dopant of F_4_-TCNQ to clarify the general applicability of the annealing approach to lowering the dark current: (a, b) Transfer characteristics: (a) PPy-BCF (b) A-PPy-BCF. (c) Transient characteristics. Note that the transfer curves were measured under *V*_d_ = −60 V and *V*_g_ from 20 to −60 V, and the photowriting (PW) was performed under 365 nm light illumination for 10 s at *V*_d_ = −60 V, whereas electrical erasing (EE) was conducted by applying *V*_g_ = −60 V for 1 s.

### Operational Parameter Dependence and Long-Term Stability of the Phototransistor Memory

The operational reliability and practical applicability of the supramolecular phototransistor memory devices were investigated using the optimized A-PPy-T25 device. The long-term retention and repeated write-read-erase-read (WRER) stability were further examined. The impact of light intensity on memory behavior was first investigated at illumination intensities ranging from 0.01 to 2.87 mW cm^–2^ at *V*_d_ = −60 V, with a writing time of 10 s; the corresponding results are presented in Figure 6a. As the light intensity increased, the programmed current gradually increased because more photocarriers were generated, thereby promoting more efficient CT-assisted charge trapping within the electret layer. Notably, even at an extremely low light intensity of 0.01 mW cm^–2^, the device still exhibited clear, distinguishable memory behavior, with a high *I*_on_/*I*_off_ current ratio of 3.96 × 10^4^. This result demonstrates the excellent photosensitivity and efficient photoinduced charge-trapping capability of the supramolecular electret system, indicating that the optimized A-PPy-T25 devices can operate reliably under ultralow-light conditions.

**Figure 6:**
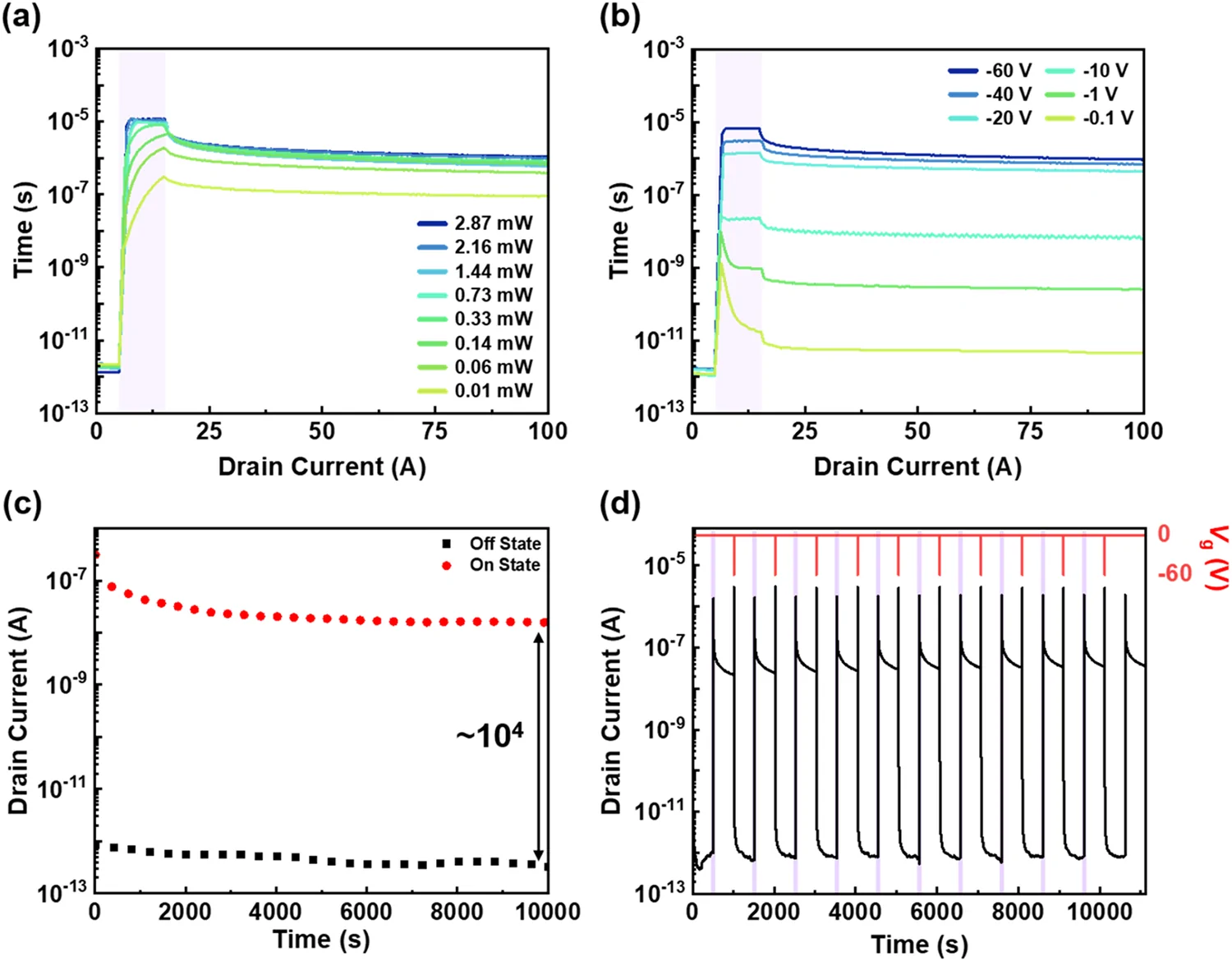
(a, b) Transient characteristics of the optimal A-PPy-T25 phototransistor memory measured under different operating conditions: (a) varying light intensities from 0.01 to 2.87 mW cm^–2^ (365 nm light illumination for 10 s, *V*_d_ = −60 V) and (b) varying reading *V*_d_ from −0.1 to −60 V (365 nm light illumination for 10 s; intensity: 1.44 mW cm^–2^). (c) Memory retention characteristics of the A-PPy-T25 phototransistor memory following photowriting and electrical erasing operations. (d) WRER cycles of the A-PPy-T25 phototransistor memory device. The reading process under the *V*_d_ = −60 V, while protowriting was performed under 365n nm light illumination for 10 s (intensity: 1.44 mW cm^–2^), and the electrical erasing was achieved by applying *V*_g_ = −60 V for 1 s.

The influence of *V*_d_ on the device operation was subsequently examined by varying *V*_d_ from −0.1 to −60 V under fixed illumination conditions (365 nm, 1.44 mW cm^–2^, 10 s), and the results are shown in Figure 6b. The devices maintained stable and distinguishable memory characteristics across the entire *V*_d_ range. As the *V*_d_ increases, the readout current and memory contrast gradually become more pronounced because the larger electric field facilitates carrier transport through the DNTT channel layer. Importantly, even at an ultralow operating voltage of −0.1 V, the device still exhibits photomemory behavior, highlighting the low-voltage operability and energy-efficient characteristics of the supramolecular phototransistor memory system. The long-term retention characteristics of the devices were further evaluated, and the results are presented in Figure 6c. The memory retention *I*_on_/*I*_off_ were programmed by 365 nm light illumination for 10 s and erased electrically by applying *V*_g_ = −60 V for 1 s. Among all investigated devices, the annealed A-PPy-T25 device exhibited the most stable retention behavior, maintaining a high *I*_on_/*I*_off_ current ratio of 4.81 × 10^4^ even after 10^4^ s. In comparison, the second-best device, PPy-T10, retained an *I*_on_/*I*_off_ ratio of only 2.26 × 10^3^ under identical testing conditions (Figure S18a, Supporting Information). Moreover, the as-cast PPy-T25 device exhibited severely degraded retention characteristics due to excessive leakage current, which substantially increased the off-state current and led to a loss of effective *I*_on_/*I*_off_ (Figure S18b, Supporting Information). These results clearly demonstrate that the annealing-induced supramolecular reorganization significantly improves the long-term operational stability of the devices by effectively suppressing leakage conduction while preserving efficient CT-assisted charge trapping pathways. To further evaluate the operational durability of the optimized devices, repeated write-read-erase-read (WRER) cycling tests were performed, and the results are shown in Figure 6d. The WRER measurements were conducted at *V*_d_ = −60 V during the reading process, while photowriting was performed under 365 nm illumination (1.44 mW cm^–2^) for 10 s, and electrical erasing was achieved by applying *V*_g_ = −60 V for 1 s. The A-PPy-T25 device maintained highly stable switching behavior throughout repeated operation cycles and maintained a large memory ratio of 3.99 × 10^4^ even after 10 consecutive WRER cycles. The excellent cycling endurance further confirms the reliable reversible operation of the supramolecular electret system. Collectively, these results demonstrate that the annealed-CT supramolecular electret system exhibits ultralow-light (0.01 mW cm^–2^) responsiveness, low-voltage operation (*V*_d_ = −1 V) capability, and outstanding long-term memory stability. The synergistic combination of efficient photoinduced intermolecular CT interactions and annealing-enhanced supramolecular organization provides a highly effective strategy for realizing stable, low-power, and high-performance organic phototransistor memory devices.

### Mechanism of the CT-Supramolecular Electret Device

Based on the optical, electrical, and interfacial characterizations discussed above, a plausible operating mechanism of the CT-supramolecular phototransistor memory device is proposed and illustrated in Figure 7. Upon light illumination, excitons are generated within the CT supramolecular electret and the DNTT channel layer. The favorable donor–acceptor energy alignment promotes photoinduced CT and charge separation. After exciton dissociation, electrons are effectively trapped in CT-assisted trapping sites formed by BCP/F_4_-TCNQ complexes, while the counter-hole carriers are transported through the DNTT channel layer, resulting in an enhanced channel current and a shifted transfer curve corresponding to the programmed ON-state. During the electrical erasing process, a negative gate bias is applied to the device, inducing hole carriers to tunnel from the DNTT channel into the supramolecular electret layer. These injected hole carriers subsequently recombine with the trapped electrons in the CT trapping sites, thereby neutralizing the stored charges and eliminating the internal electric field generated during photowriting. Beyond neutralizing trapped electrons, the negative bias further facilitates the trapping of excess holes within the electret matrix. Consequently, the combination of electron eradication and sequential hole storage successfully drives the channel current back to the OFF state, completing the memory operation cycle.

**Figure 7:**
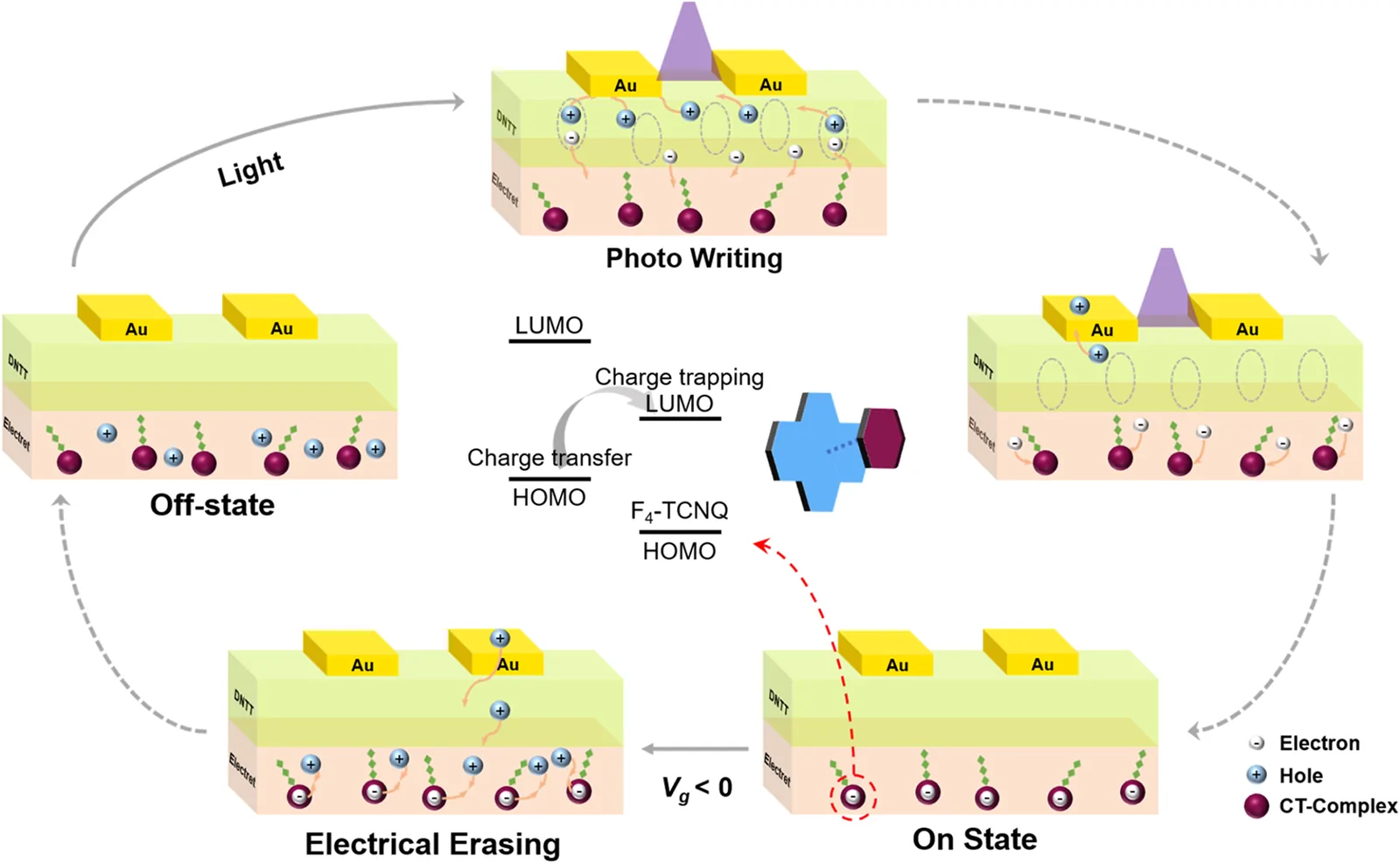
Proposed operating mechanism of the CT-supramolecular phototransistor memory device. (i) Photowriting process: UV illumination induces intermolecular charge transfer between the pyrene donor units and F_4_-TCNQ acceptors, facilitating charge separation and generating trapped charges within the supramolecular electret layer, thereby generating the memory ON state. (ii) Electrical erasing process: the application of a negative gate bias releases the trapped charges from the electret layer and promotes charge redistribution within the supramolecular system, switching the device to the OFF state and enabling reversible memory operation.

## Conclusion

In summary, carbohydrate-based BCPs, PPy and PB, were doped with F_4_-TCNQ to construct a series of supramolecular electret systems for organic phototransistor memory applications. Optical characterization revealed significant photoluminescence quenching and shorter fluorescence lifetimes upon F_4_-TCNQ incorporation, confirming efficient photoinduced intermolecular CT in the PPy/F_4_-TCNQ systems. Owing to the more favorable energy-level alignment between pyrene and F_4_-TCNQ, the PPy-based devices exhibited substantially superior charge-trapping capability and memory performance compared with the PB-based counterparts. More importantly, the sequential solvent-vapor and thermal annealing treatments significantly improved dopant accommodation within the BCP matrix, enabling stable device operation even at high F_4_-TCNQ loading. As a result, the optimized A-PPy-T25 device exhibited high photosensitivity of 1.27 × 10^7^, *I*_on_/*I*_off_ ratio of 1.61 × 10^6^, excellent retention characteristics with a memory ratio of 4.81 × 10^4^ after 10^4^ s, and stable WRER operation with a memory ratio of 3.99 × 10^4^ after 10 cycles. Furthermore, reliable memory operation was maintained under low-light-intensity and low-operating-voltage (−0.1 V) conditions. These findings demonstrate that carbohydrate-based BCPs can serve as effective supramolecular hosts to regulate CT interactions and dopant accommodation, offering a versatile molecular design strategy for developing high-performance supramolecular optoelectronic memory devices. Future studies may further explore different carbohydrate architectures, CT-active molecular combinations, and supramolecular electret designs to realize next-generation low-power, high-density organic photomemory systems.

## Experimental Section

### Materials

The synthetic procedures of maltotriose-*block*-poly­(1-pyrenemethyl methacrylate) (PPy, *M_n_* = 15,000, PDI = 1.21) and maltotriose-*block*-poly­(benzyl methacrylate) (PB, *M_n_* = 4400, PDI = 1.29) were presented in the Supporting Information, including ^1^H NMR, FT-IR, and GPC analyses were shown in Figures S1–S8 (Supporting Information). Anhydrous dimethylformamide (99.8%) was purchased from Sigma-Aldrich. 2,3,5,6-Tetrafluoro-7,7,8,8-tetracyanoquinodimethane (F_4_-TCNQ) was obtained from Ultra Fine Chemical Technology Corp. Dinaphtho­[2,3-b:2′,3′-*f*]­thieno­[3,2-*b*]­thiophene (DNTT) was obtained from Luminescence Technology Corp. All chemicals were used as received without further purification.

### Fabrication and Characterization of Phototransistor Memory Devices

To formulate the CT supramolecular solutions, the block copolymers (PPy or PB) and F_4_-TCNQ were separately dissolved in DMF at a concentration of 20 mg mL^–1^. Subsequently, the solutions were mixed at predetermined molar ratios to obtain F_4_-TCNQ contents of 5 mol % (T5), 10 mol % (T10), and 25 mol % (T25) with respect to the repeating units of the polymer matrices (Table S1, Supporting Information). Finally, the resulting supramolecular solutions were stirred overnight at room temperature to ensure homogeneous mixing before film fabrication. The highly n-doped Si substrates with a 100 nm-thick SiO_2_ layer were treated with a UV-ozone cleaner to enhance surface hydrophilicity. The supramolecular solutions were filtered through a 0.22 μm PTFE filter to remove aggregates and then spin-coated at 800 rpm for 60 s. For the annealed group, the spin-coated supramolecular films were first subjected to solvent-vapor annealing at room temperature for 24 h. The films were placed in a sealed 2 L glass container with an open 20 mL vial filled with 5 mL of a mixed solvent (THF:H_2_O = 1:1, v/v). Subsequently, the samples were thermally annealed at 140 °C for an additional 24 h. Hereafter, devices subjected to the sequential solvent vapor and thermal annealing treatments are denoted as annealed devices (A-PB and A-PPy denotations), whereas devices fabricated without these post-treatments are referred to as as-cast devices. Next, a 50 nm-thick DNTT channel layer and 70 nm-thick Au source/drain electrodes were sequentially deposited via thermal evaporation at a base pressure of 10^–7^ Torr. The Au electrodes were patterned through a mask, defining a channel length (*L*) of 50 μm and a width (*W*) of 1000 μm. The electrical characteristics of the memory devices were measured using a Keithley 4200-SCS semiconductor parameter analyzer within a nitrogen-filled glovebox to ensure an inert atmosphere. Transfer characteristics were recorded at a constant *V*_d_ = −60 V, with the *V*_g_ sweeping from 20 to −60 V. The field-effect mobility (μ_h_) and Δ*V*_th_ were extracted from the saturation regime of the transfer curves using the following equation

$$I_{DS}=\mu_{h}\frac{W}{2L}C_{\mathrm{ox}}{\left(V_{g}-V_{th}\right)}^{2}$$

where *C*_ox_ denotes the areal capacitance (34.5 nF cm^–2^) of the 100 nm thick SiO_2_. For the phototransistor memory evaluation, the photowriting process was performed by illuminating the device with a 365 nm UV light source (Titan Electro-Optics Co., Ltd., 0.01–2.87 mW cm^–2^) for 10 s at *V*_g_ = 0 V under *V*_d_ = −60 V. The light intensity was calibrated using a StarLite power meter. The electrical erasing process was subsequently performed by applying a negative gate-bias pulse of −60 V for 1 s at *V*_d_ = 0 V.

### Characterizations

^1^H NMR spectra were recorded on a Bruker AVIII 500 MHz FT-NMR spectrometer utilizing deuterated chloroform (CDCl_3_) as the solvent. Fourier-transform infrared (FT-IR) spectra were obtained using a PerkinElmer Spectrum Two FT-IR spectrometer. Ultraviolet–visible (UV–vis) absorption spectra were measured on a Hitachi U-4100 spectrophotometer. Cyclic voltammetry (CV) measurements were carried out using a CHI 6273E electrochemical analyzer to determine the HOMO energy levels of the block copolymers. The optical bandgaps were estimated from the absorption onset of the UV–vis spectra, and the corresponding LUMO levels were subsequently calculated from the HOMO energy and optical bandgap. Steady-state photoluminescence (PL) spectra were collected using a HORIBA Jobin Yvon Fluorolog-3 spectrofluorometer. Time-resolved photoluminescence (TRPL) decays were recorded on the same Fluorolog-3 system under a 375 nm laser excitation. The decay profiles were fitted using a biexponential function

$$I\left(t\right)=A_{1}e^{-t/\tau_{1}}+A_{2}e^{-t/\tau_{2}}$$

where *A*_1_ and *A*_2_ are the amplitudes of the fast and slow decay components, respectively, and τ_1_ and τ_2_ are the corresponding fluorescence lifetimes. The intensity-averaged lifetime (τ_avg_) was calculated according to

$$\tau_{\mathrm{avg}}=\frac{A_{1}{\tau_{1}}^{2}+A_{2}{\tau_{2}}^{2}}{A_{1}\tau_{1}+A_{2}\tau_{2}}$$

The capacitance of the electret layer was determined by capacitance measurements using a metal–insulator–metal (MIM) capacitor structure at a frequency of 1 kHz. The MIM capacitor consisted of Au/electret/100 nm SiO_2_/highly doped Si, where the Au top electrode had an active area of 500 × 500 μm^2^. Since the measured capacitance corresponds to the series combination of the SiO_2_ dielectric and the electret layer, the capacitance of the electret (*C*_electret_) was obtained according to

$$\frac{1}{C_{\mathrm{total}}}=\frac{1}{C_{{\mathrm{SiO}}_{2}}}+\frac{1}{C_{\mathrm{electret}}}$$

where *C*_total_ is the experimentally measured capacitance and *C*_SiO_2__ is the capacitance of the 100 nm SiO_2_ dielectric layer. The capacitance per unit area of the electret was then calculated by

$$C_{i}=\frac{C_{\mathrm{electret}}}{A}$$

where *A* is the effective capacitor area defined by the Au top electrode. The areal trap density (Δ*N*) was subsequently estimated from the threshold voltage shift between the photowritten and electrically erased states according to

$$\Delta N=\frac{C_{i}\cdot {\Delta V}_{\mathrm{th}}}{e}$$

where Δ*V*_th_ is the threshold voltage shift and *e* = 1.602 × 10^–19^ C is the elementary charge. The calculated Δ*N* represents the density of trapped charges per unit area within the electret layer. The surface morphology and roughness of the electret films were investigated via atomic force microscopy (AFM, Bruker MultiMode 8) operating in tapping mode. Contact angle measurements were performed on a CAM-110 instrument (Creating Nano Technologies, Inc.) using deionized water and diiodomethane as the probing liquids to determine the total surface energy and its polar and dispersive components. The total surface energy and its dispersive and polar components were calculated using the Owens–Wendt model

$$\gamma_{L}\left(1+\cos\theta \right)=2\left(\sqrt{\gamma_{S}^{D}\gamma_{L}^{D}}+\sqrt{\gamma_{S}^{P}\gamma_{L}^{P}}\right)$$

where θ is the contact angle, γ_S_ and γ_L_ are the surface energies of the solid and liquid, respectively, and the superscripts D and P represent the dispersive and polar components.

## Supplementary Material



## Data Availability

Data will be made available on request.

## Notes

Syntheses and structure characterizations of the materials; optical and electrochemical characterization data of the supramolecular systems, including UV–vis absorption spectra and CV profiles; tables of polymer properties, contact angles, surface energy components, and photomemory performance parameters, and a performance comparison with previously reported literature; transfer and memory characteristics of the DNTT-based devices; C–V curves; and AFM images along with roughness summaries (PDF)
