# Supplementary material for: Boosting the Dopant Accommodation in Charge-Transfer Supramolecular Electrets Using Carbohydrate Block Copolymers in Phototransistor Memory
Source: ACS Omega. 2026 Aug 17;11(34):52014–28. doi: 10.1021/acsomega.6c07010 (PMC13625098; doi:10.1021/acsomega.6c07010)
Supplement: Supplementary file 1 [file ao6c07010_si_001.pdf]

Supporting Information

Boosting the Dopant Accommodation in Charge-Transfer Supramolecular Electrets Using Carbohydrate Block Copolymers in Phototransistor Memory

Ching-Wei Yang,[a] *Qi-An Hong,*[b] *Yi-Hsun Weng,*[b] *Ping-Jui Yu,*[b] *Bi-Hsuan Lin,*[c]

Cheng-Liang Liu,[d,e] *Yan-Cheng Lin,*[f,e*] *Wen-Chang Chen*[a,b,e*]

[a] Institute of Polymer Science and Engineering, National Taiwan University, Taipei 10617, Taiwan

[b] Department of Chemical Engineering, National Taiwan University, Taipei 10617, Taiwan

[c] National Synchrotron Radiation Research Center, Hsinchu 300092, Taiwan

[d] Department of Materials Science and Engineering, National Taiwan University, Taipei 10617, Taiwan.

[e] Advanced Research Center of Green Materials Science and Technology, National Taiwan University, Taipei 10617, Taiwan.

[f] Department of Chemical Engineering, National Cheng Kung University, Tainan 70101, Taiwan

*Corresponding authors. E-mail: ycl@gs.ncku.edu.tw (Y.-C. L.), chenwc@ntu.edu.tw (W.-C. C.)

Table S1. Mole fractions of F_4-TCNQ in supramolecular electrets

	**Mole fraction of F_4-TCNQ (mol%)**
**T0**	0
**T5**	5
**T10**	10
**T25**	25

Table S2. Summary of steady-state PL quenching efficiency and TRPL average carrier lifetimes decrement. Note that the PL quenching efficiency is derived from the integral of the steady-state emission profiles.

	**PL Quenching Efficiency (%)**	**Lifetime Decrement (%)**
**PPy-T0**	0	0
**PPy-T5**	51	76
**PPy-T10**	82	80
**PPy-T25**	96	100
**A-PPy-T0**	0	0
**A-PPy-T5**	39	68
**A-PPy-T10**	53	70
**A-PPy-T25**	69	79

Table S3. Fitted parameters of photoluminescence decay traces for the supramolecular electret films with different F_4-TCNQ doping concentrations

	A_1	τ_1 **(ns)**	A_2	τ_2 **(ns)**	τ_{avg} **(ns)**
**PPy-T0**	0.940	5.196	0.060	23.721	9.393
**PPy-T5**	0.999	2.185	0.001	9.023	2.214
**PPy-T10**	0.650	1.691	0.350	2.251	1.925
**PPy-T25**	-	-	-	-	-
**A-PPy-T0**	0.952	5.311	0.048	26.173	9.498
**A-PPy-T5**	0.996	2.881	0.004	12.464	3.052
**A-PPy-T10**	0.998	2.734	0.002	13.888	2.840
**A-PPy-T25**	0.999	2.008	0.001	8.157	2.024

Table S4. Capacitance parameters and extracted trap densities of the PB- and PPy-based supramolecular electrets before and after annealing.

	**Total capacitance [pF]**	**Electret capacitance [pF]**	$C_{i,\text{ electret}}$ **[nF/cm^2]**	V_{th} **[V]**	**Trap density [×10^{12} cm^{-2}]**
**PB-T0**	35.2	59.6	23.8	5.3	0.8
**PB-T5**	34.9	58.5	23.4	19.3	2.8
**PB-T10**	34.6	57.9	23.1	31.7	4.6
**PB-T25**	35.5	60.2	24.1	19.1	2.9
**A-PB-T0**	35.1	59.2	23.7	4.7	0.7
**A-PB-T5**	34.9	58.6	23.4	8.6	1.3
**A-PB-T10**	34.9	58.6	23.4	10.2	1.5
**A-PB-T25**	35.6	60.5	24.2	29.0	4.4
**PPy-T0**	34.1	56.3	22.5	50.4	7.1
**PPy-T5**	35.6	60.7	24.3	54.7	8.3
**PPy-T10**	35.3	59.8	23.9	56.7	8.5
**PPy-T25**	35.8	61.3	24.5	35.9	5.5
**A-PPy-T0**	34.6	57.8	23.1	47.2	6.8
**A-PPy-T5**	34.7	58.0	23.2	54.7	7.9
**A-PPy-T10**	35.4	59.9	24.0	49.4	7.4
**A-PPy-T25**	35.5	60.3	24.1	36.7	5.5

Table S5. Device characteristics of organic phototransistor memory based on 5 mol% and 10 mol% F_4-TCNQ supramolecular electrets.

Electret	μ[a] [cm^2 V^{-1} s^{-1}]	ΔV_{th}[b] [V]	I_{on} / I_{off}[c]	Photosensitivity[d]	Retention ratio[e] (%)
**PB-T5**	0.18	19.3	1.32×10^1	2.18×10^4	8.2
**A-PB-T5**	0.17	8.6	2.09×10^2	6.45×10^4	7.3
**PB-T10**	0.15	31.7	1.00×10^4	1.37×10^5	0.3
**A-PB-T10**	0.25	10.2	5.28×10^2	2.37×10^4	0.1
**PPy-T5**	0.17	54.7	1.51×10^6	6.60×10^6	22.9
**A-PPy-T5**	0.21	54.7	3.88×10^5	3.63×10^5	26.7
**PPy-T10**	0.17	56.7	1.58×10^6	1.14×10^7	13.0
**A-PPy-T10**	0.13	49.4	1.45×10^5	3.13×10^6	41.8

[a] Mobility obtained from the saturation regime of the initial transfer characteristics. Power of light sources: 365 nm, 1.44 mW cm^{-2}. [b] Memory window: $\Delta V_{th} = V_{th,write} - V_{th,erase}$. [c] The ratio of photocurrent to dark current. [d] Photosensitivity: $P = (I_{on} - I_{off})/ I_{off}$. [e] Retention ratio was defined as the photocurrent at 100 s divided by that at 1 s after photowriting. Retention ratio (%) = $I_1 / I_{100} \times 100$ (%)

Table S6. Comparison of representative organic phototransistor memories employing different charge-storage layers

Type	Charge-storage layer	Channel layer	Operation	I_{on}/I_{off} [a]	Ref.
**Floating gate**	$MAPbBr_3$/P2VP	BPE-PTCDI	530 nm, 10 mW cm^{-2}, 60 s	~10^4	S-1
**Floating gate**	$MAPbBr_3$/PS-b-PEO	P3HT	520 nm, 454 mW cm^{-2}, 240 s	1.51×10^4	S-2
**Small molecule**	APTS/C_{60} SAM	Pentacene	650 nm, $V_g = +50V$, 20 min	~10^3	S-3
**Small molecule**	PDI-Sol4	BPE-PDI	405 nm, 10 mW cm^{-2}, 300 s	3.4×10^4	S-4
**Electret**	PF/PS	Pentacene	405 nm, 5 mW cm^{-2}, 1 s	3.1×10^6	S-5
**Electret**	PAMA	P3HT	450 nm, 30 mW cm^{-2}, 60 s	~10^5	S-6
**Electret**	PTPA-CNBr	Pentacene	395–415 nm, 5 mW cm^{-2},0.1 ms	~10^6	S-7
**Electret**	PVPh:TCPP	DNTT	450 nm, 20 mW cm^{-2}, 5s	1.12×10^8	S-8
**Electret**	TPA-CN-TPE	Pentacene	365 nm, 720 μW cm^{-2}, 1 s	~10^4	S-9
**Electret**	PPy/F_4-TCNQ	DNTT	365 nm. 1.44 mW cm^{-2}, 10 s	1.61×10^6	This work

[a] The ratio of photocurrent to dark current.

Materials.

Maltotriose, 1-pyrenylmethyl methacrylate (PyMA, >99.0%), and acetic anhydride were purchased from Sigma-Aldrich. Propargylamine and benzyl methacrylate (BMA, >98.0%) were acquired from Tokyo Chemical Industry (TCI). Initiators, catalysts, and related reagents, including ethyl α-bromoisobutyrate, 2-azidoethyl α-bromoisobutyrate, sodium azide, *N*,*N*,*N*',*N*'',*N*''-pentamethyl diethylenetriamine (PMDETA), and copper(I) bromide (CuBr), were also purchased from Sigma-Aldrich. All solvents, including *N*,*N*-dimethylformamide (DMF), dichloromethane (DCM), methanol (MeOH), toluene, tetrahydrofuran (THF), and anisole, were supplied by Sigma-Aldrich.

Synthesis of N-maltotriose-3-acetamido-1-propyne (MT-alkyne).

Maltotriose (5 g, 9.90 mmol) and propargylamine (12.67 mL, 198 mmol) were introduced into a round-bottom flask and stirred at room temperature under a nitrogen atmosphere for 72 h. The reaction mixture was then precipitated into DCM to remove the unreacted propargylamine. Subsequently, the obtained precipitate was dissolved in a mixed solvent of acetic anhydride and MeOH (v/v = 1:10, 440 mL). The solution was stirred at room temperature for 48 h, and the completion of the acetylation was confirmed by thin-layer chromatography (TLC). Excess acetic anhydride was removed via co-evaporation using a toluene/MeOH mixture. Finally, the crude product was precipitated into DCM and dried in a vacuum oven at 60 °C overnight to afford MT-alkyne as a white solid (3.32 g, 66.4% yield). ^{1}H NMR (500 MHz, D_2O, **Figure S1**): δ (ppm) 5.57 and 5.11 (rotamers, 1H, –C**H**NAc), 5.43–5.39 (m, 2H, H-1), 4.3–3.3 (m, H-2, -3, -4, -5, -6), 2.76 and 2.60 (t, 1H, rotamers, –CH_2C≡C**H**), 2.34 and 2.26 (s, rotamers, 3H, -C**H$_3$**).

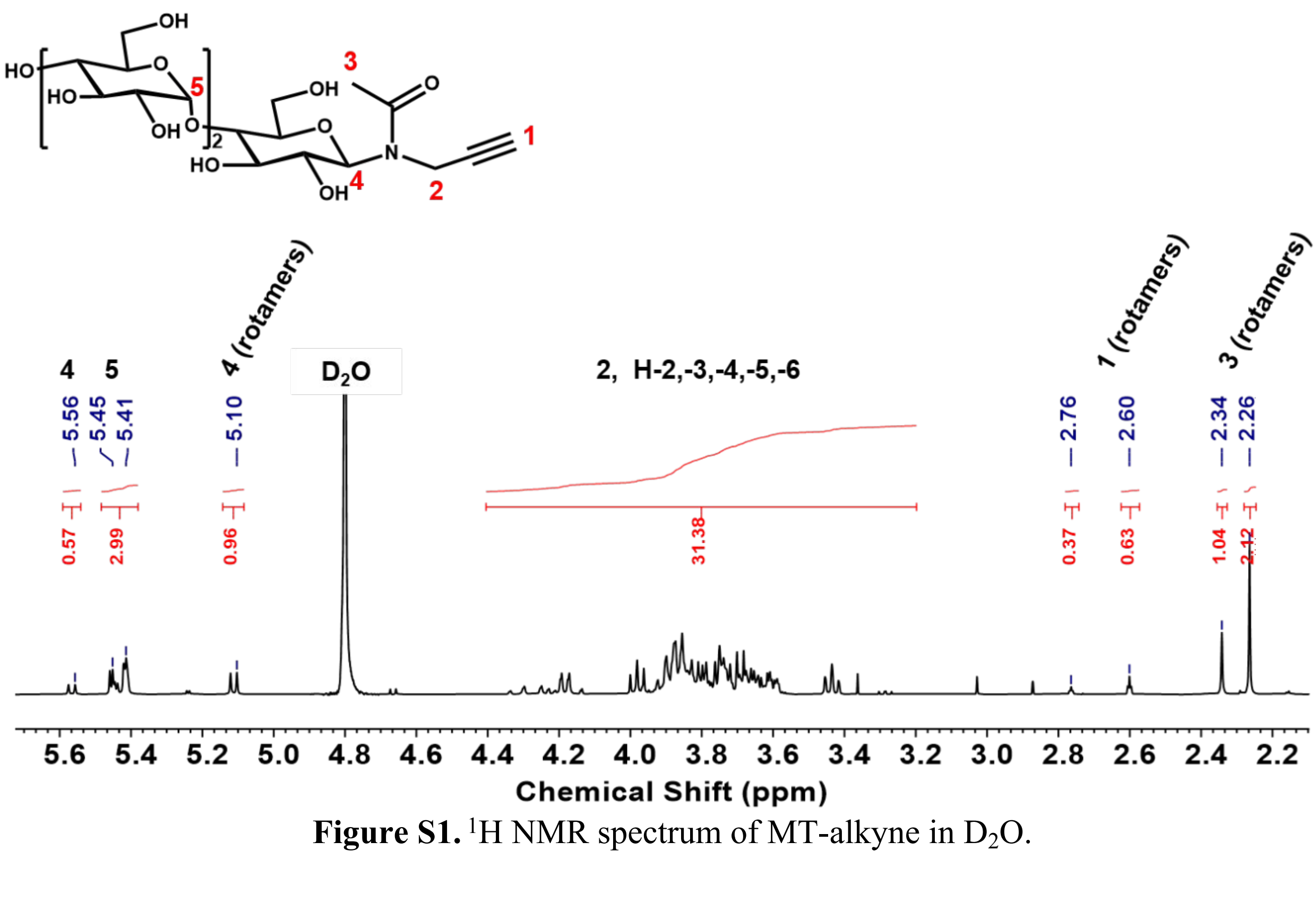

Figure S1. ^{1}H NMR spectrum of MT-alkyne in D_2O.

Synthesis of azido-functionalized poly(benzyl methacrylate) (PBMA-N_3).

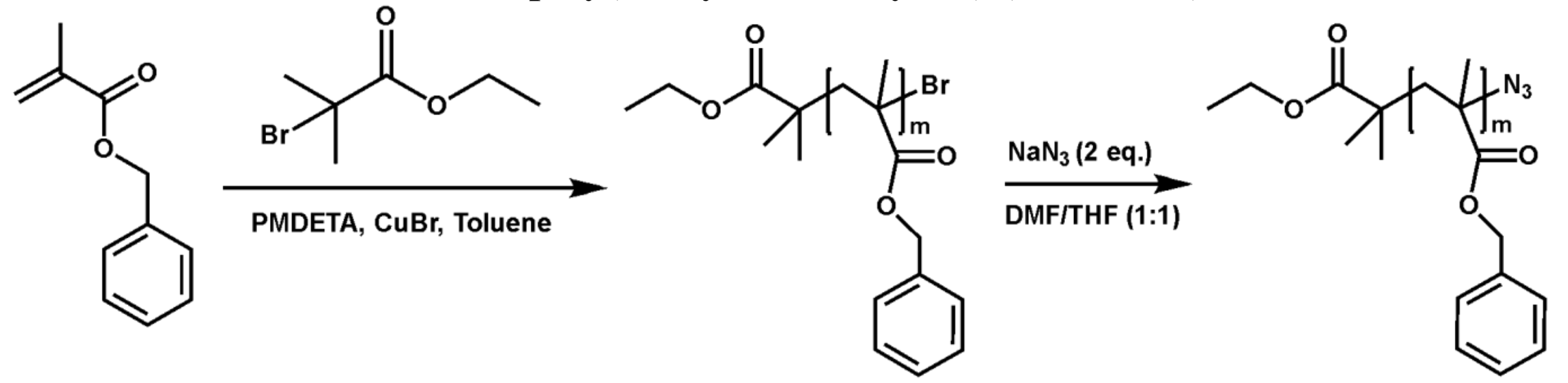

Bromine-terminated PBMA (PBMA-Br) was synthesized via atom-transfer radical polymerization (ATRP). Briefly, BMA (3 g, 17.03 mmol), ethyl α-bromoisobutyrate (0.34 mmol), PMDETA (0.34 mmol), and toluene (17 mL) were degassed via three freeze-pump-thaw cycles and transferred into a nitrogen-purged Schlenk flask containing pre-purified CuBr (0.34 mmol). After reacting at 67 °C for 1.5 h, the mixture was air-quenched, passed through neutral alumina, and precipitated into cold MeOH. Vacuum drying at 60 °C yielded PBMA-Br as a white solid (1.08 g, 36%). Size-exclusion chromatography revealed an M_n of 4405 g/mol and PDI of 1.25. ^{1}H NMR (500 MHz, d_8-THF, **Figure S2**): δ (ppm) 7.30 (m, Ar-**H**, 5H), 4.91 (d, -C**H**$_2$-, 4H), 1.88 (m, -C**H**$_2$-, 2H), 1.40 (-C**H**$_3$, 3H), 1.29 (-C**H**$_3$, 3H), 0.96 (s, -C**H**$_3$, 3H), 0.80 (s, -C**H**$_3$, 3H).

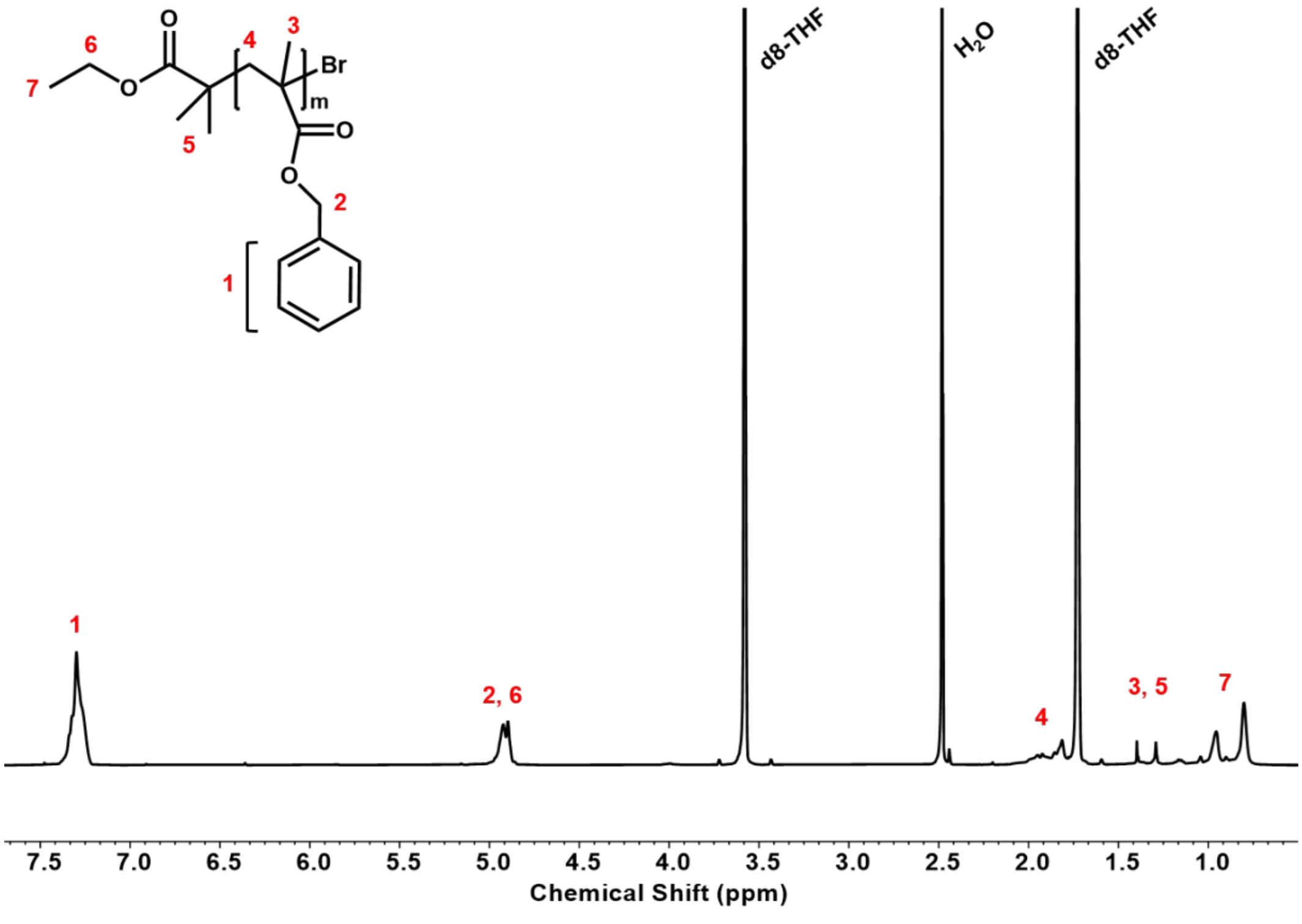

Figure S2. ^{1}H NMR spectrum of PBMA-Br in d_8-THF.

Subsequently, to introduce the azide functionality, PBMA-Br (800 mg, 0.18 mmol) was reacted with sodium azide (23.6 mg, 0.36 mmol) in a DMF/THF mixture at 65 °C overnight under nitrogen. The crude mixture was extracted with chloroform/water and then dried under reduced pressure to obtain PBMA-N_3 in 95% yield (752 mg). ^{1}H NMR (500 MHz, d_8-THF, **Figure S3**): δ (ppm) 7.30 (m, Ar-**H**, 5H), 4.91 (d, -C**H**$_2$-, 4H), 1.87 (m, -C**H**$_2$-, 2H), 1.40 (s, -C**H**$_3$, 3H), 1.29 (s, -C**H**$_3$, 3H), 0.95 (s, -C**H**$_3$, 3H), 0.80 (s, -C**H**$_3$, 3H).

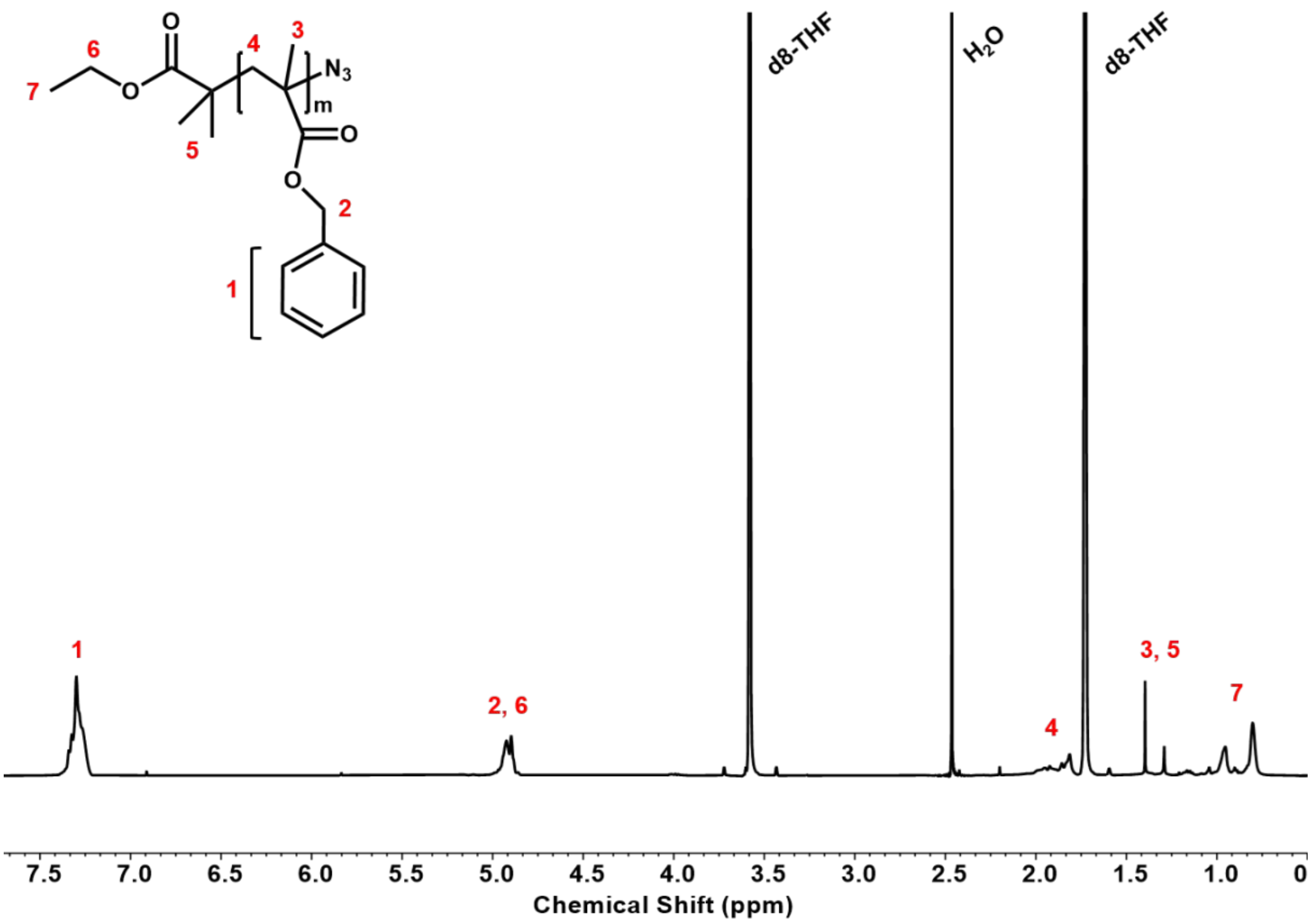

Figure S3. ^{1}H NMR spectrum of PBMA-N_3 in d_8-THF.

Synthesis of azido-functionalized poly(1-pyrenemethyl methacrylate) (PPyMA-N_3).

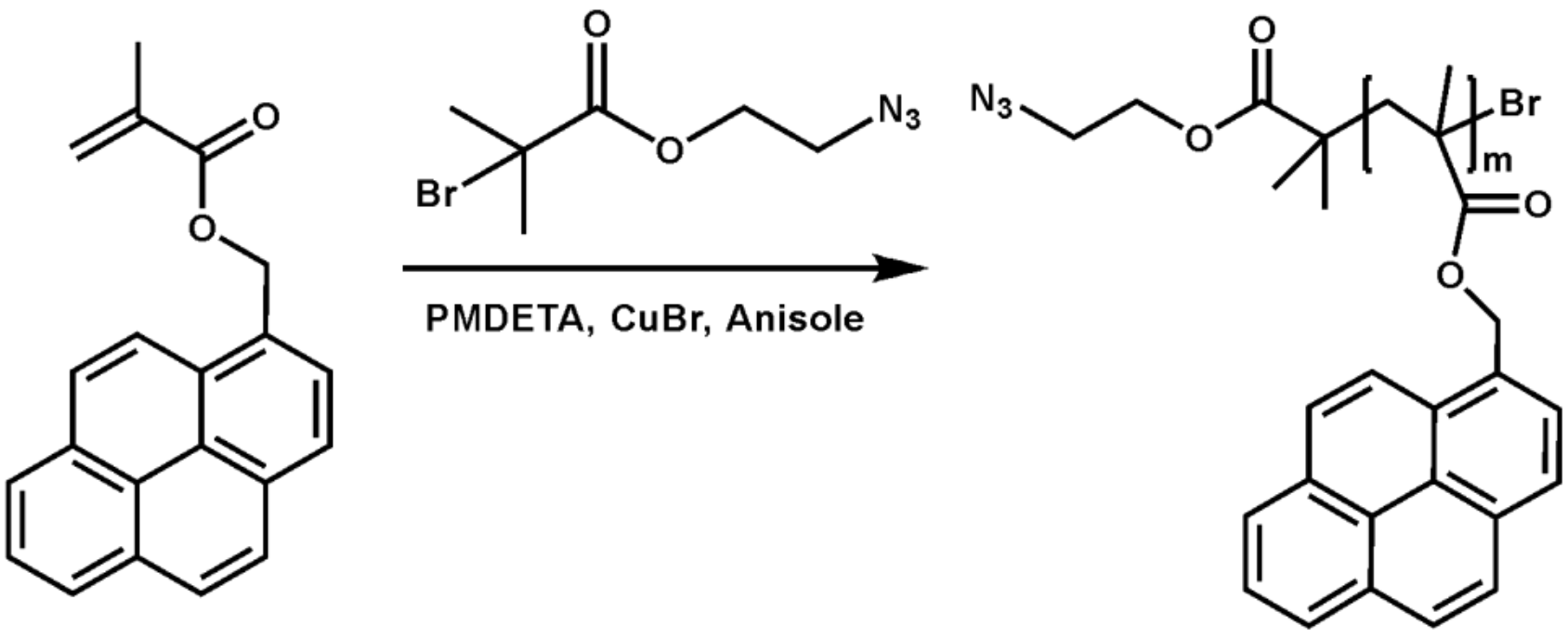

The 1-pyrenylmethyl methacrylate monomer (PyMA, 0.5 g, 1.67 mmol), the initiator 2-azidoethyl α-bromoisobutyrate (0.02 mmol), PMDETA (0.02 mmol), and 0.9 M anisole were degassed via three freeze-pump-thaw cycles and transferred into a nitrogen-purged Schlenk flask containing pre-purified CuBr (0.02 mmol). After reacting at 70 °C for 0.5 h, the mixture was air-quenched, diluted with THF, passed through neutral alumina, and precipitated into cold MeOH. Vacuum drying at 60 °C yielded PPyMA-N_3 as a white solid (0.29 g, 58%). Size-exclusion chromatography revealed an M_n of 14,300 g/mol and PDI of 1.20. ^{1}H NMR (500 MHz, $CDCl_3$, **Figure S4**): δ (ppm) 7.54 (m, Ar-**H**, 9H), 5.29-5.05 (d, -C**H**$_2$-, 2H), 4.25 (-OC**H**$_2$-, 2H), 2.35 (-C**H**$_2$-, 2H), 1.77 (m, -C**H**$_2$-, 2H), 0.87 (s, -C**H**$_3$, 9H).

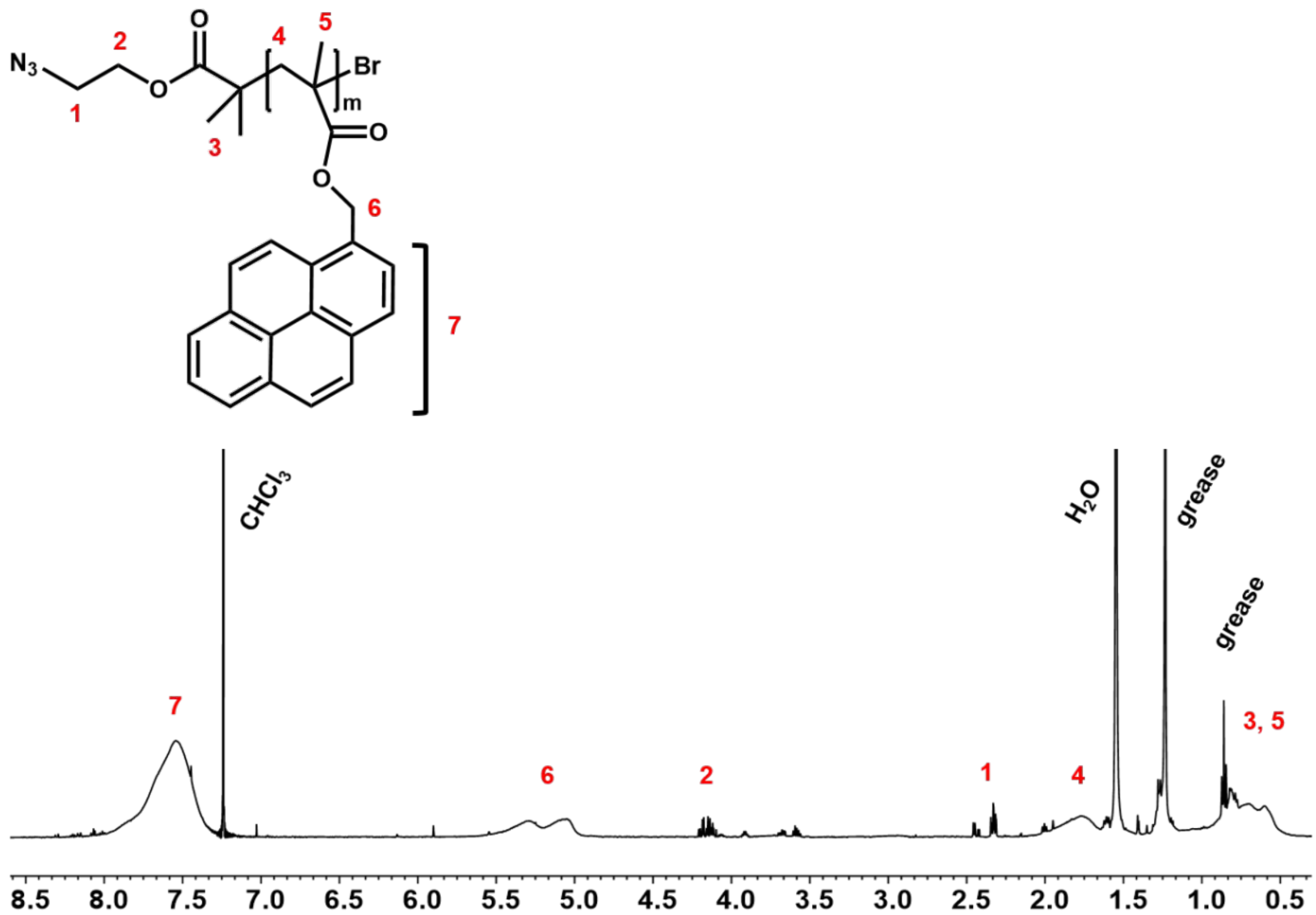

Figure S4. ^{1}H NMR spectrum of PPyMA-N_3 in C*d*Cl_3.

Synthesis of MT-*b*-PArMA.

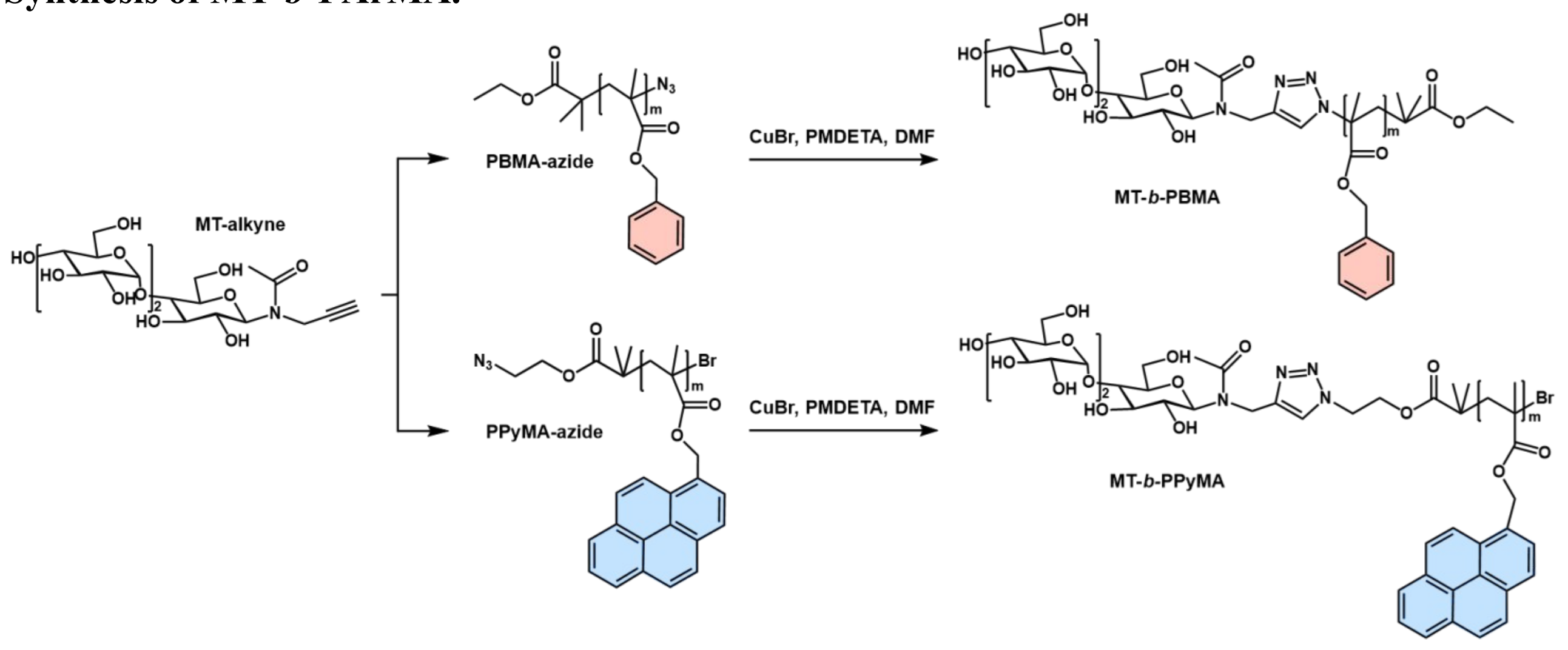

Block copolymer (MT-*b*-PBMA and MT-*b*-PPyMA) was synthesized via a copper-catalyzed azide-alkyne cycloaddition (CuAAC) click reaction. Briefly, the azide-functionalized polymer (PBMA-N_3, 300 mg; PPyMA-N_3, 200 mg, 1 eq.), MT-alkyne (85.3 mg for MT-*b*-PBMA; 28.43 mg for MT-*b*-PPyMA, 2 eq.), and PMDETA (5 eq.) were dissolved in 0.01 M DMF. The mixture was purged with nitrogen for 1 h. Subsequently, CuBr (2 eq.) was added, followed by vacuum-nitrogen cycles to ensure a strictly anaerobic environment. The click reaction proceeded at 65 °C in an oil bath under a nitrogen atmosphere for a specific duration (24 h for MT-*b*-PBMA and 72 h for MT-*b*-PPyMA). The crude mixture was diluted with THF, passed through a neutral alumina column, and precipitated into cold MeOH. Subsequent dialysis (MWCO = 2000 Da) and vacuum drying afforded the target block copolymers as white solids. The final yields were 271.7 mg (79%) for MT-*b*-PBMA and 105 mg (50%) for MT-*b*-PPyMA. Molecular weight evaluated by size-exclusion chromatography (SEC): MT-*b*-PBMA, M_n = 4,400, PDI = 1.29 (^{1}H-NMR, 500 MHz, $CDCl_3$, **Figure S5**) and MT-*b*-PPyMA, M_n =15,000, PDI = 1.21 (^{1}H-NMR, 500 MHz, $CDCl_3$, **Figure S6**).

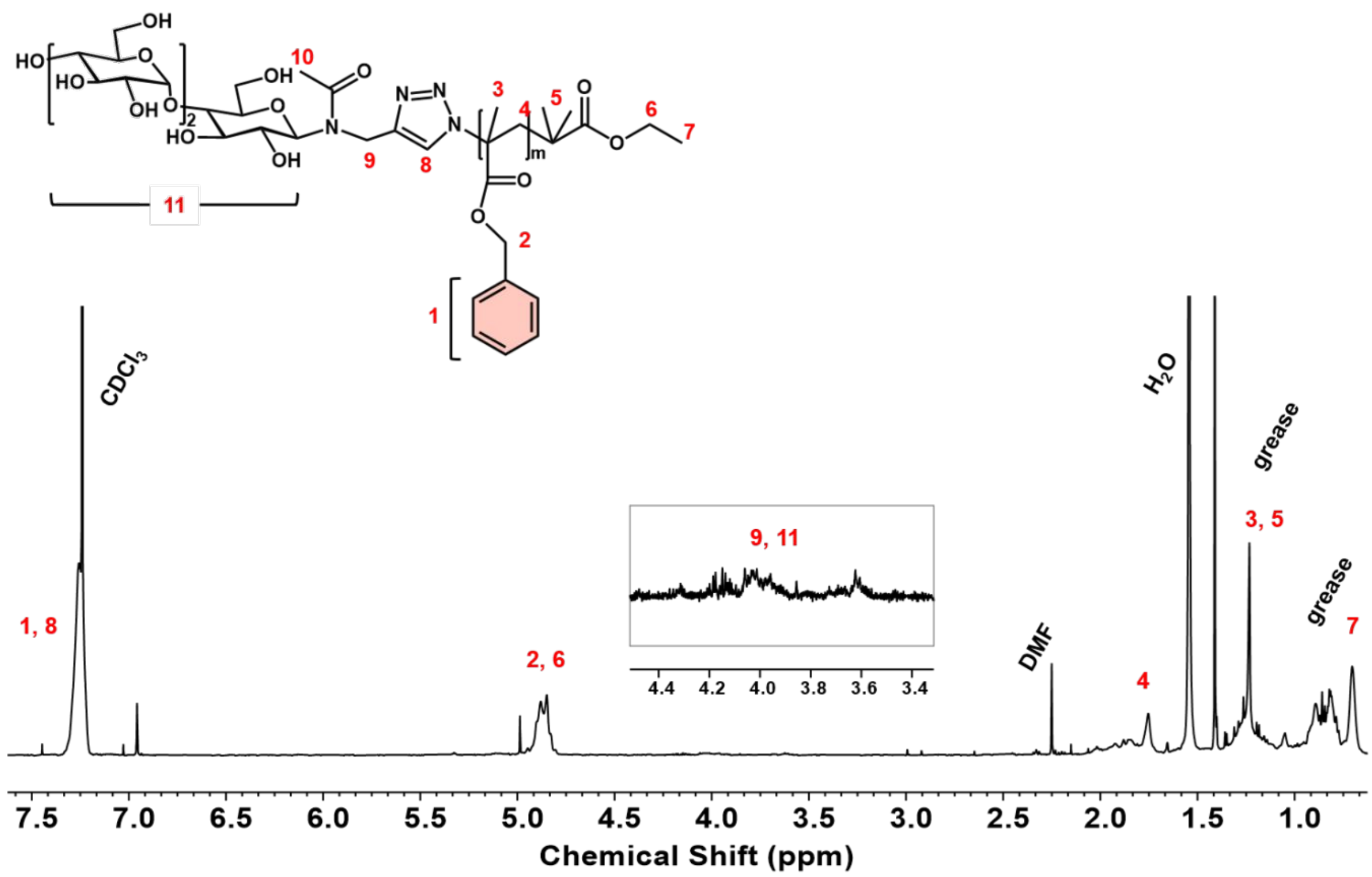

Figure S5. ^{1}H NMR spectrum of MT-*b*-PBMA (PB) in $CDCl_3$.

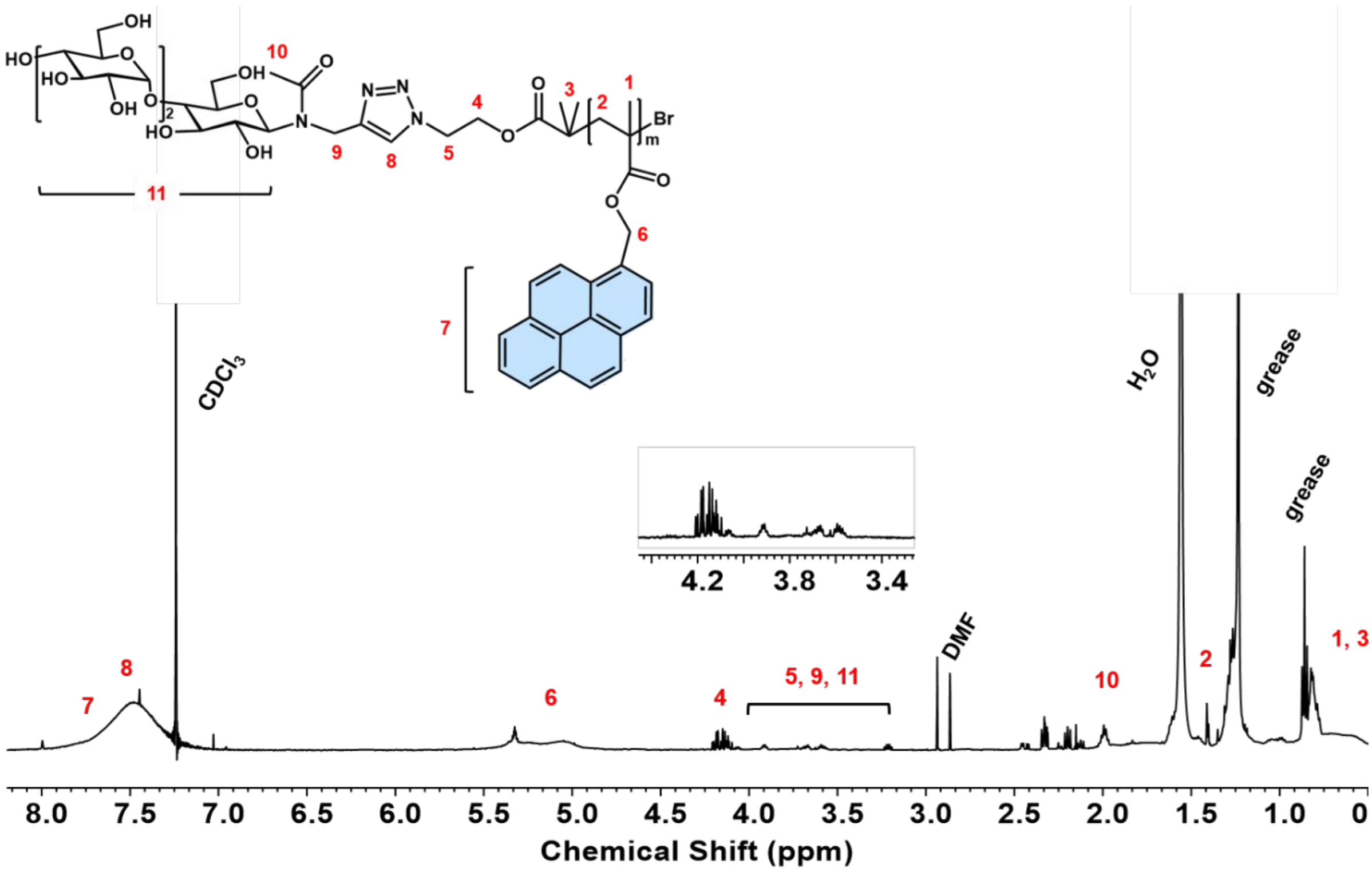

Figure S6. ^{1}H NMR spectrum of MT-*b*-PPyMA (PPy) in $CDCl_3$.

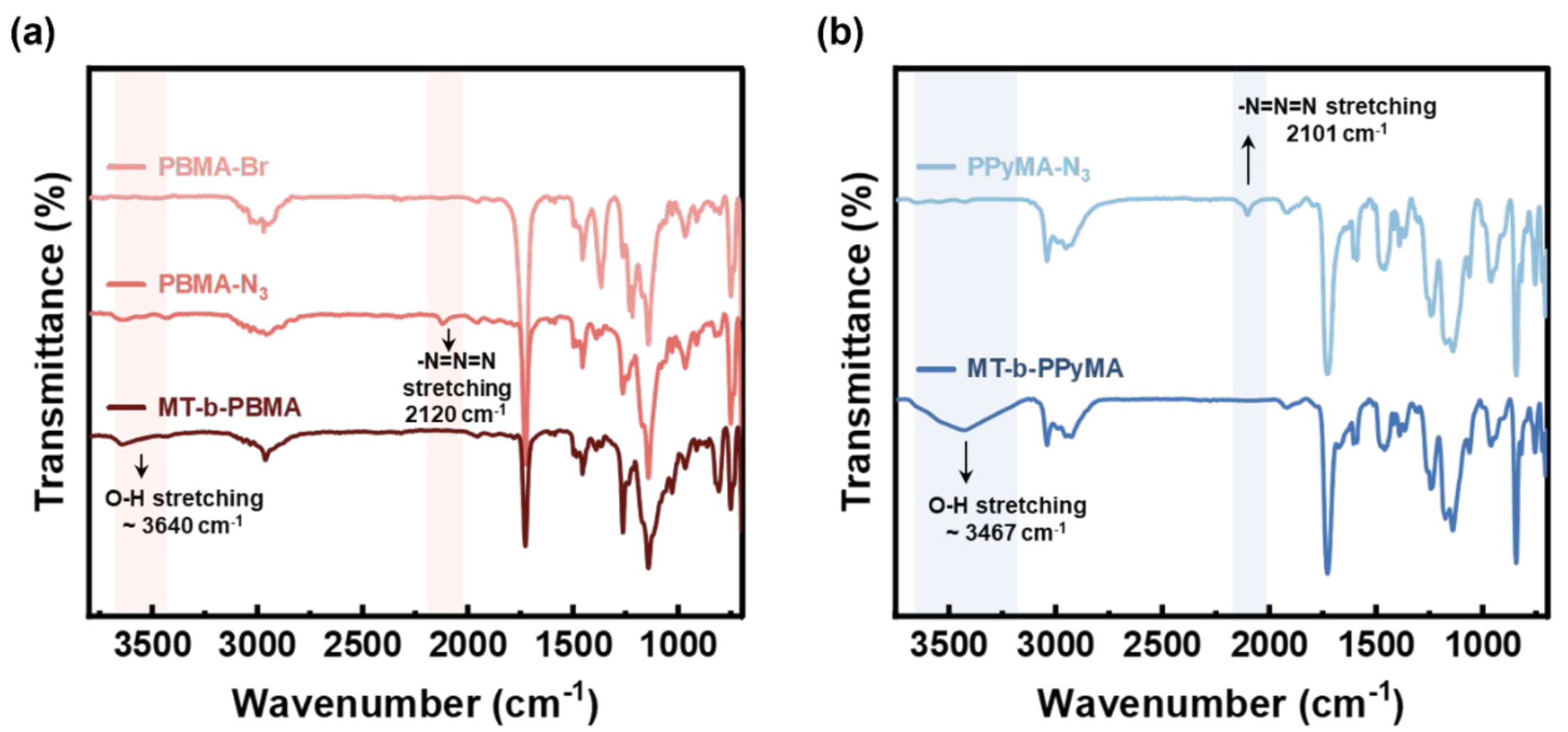

Figure S7. FT-IR spectra demonstrating successful synthesis via click chemistry. (a) Conversion of PBMA-Br to PBMA-N_3 and subsequent conjugation to form MT-*b*-PBMA and (b) comparison between PPyMA-N_3 and MT-*b*-PPyMA. The complete disappearance of the characteristic azide (-N_3) stretching peak at ~ 2120 cm^{-1} accompanied by the emergence of a broad O-H stretching band ~ 3467 cm^{-1} indicates the successful consumption of azide groups and the incorporation of carbohydrate moieties.

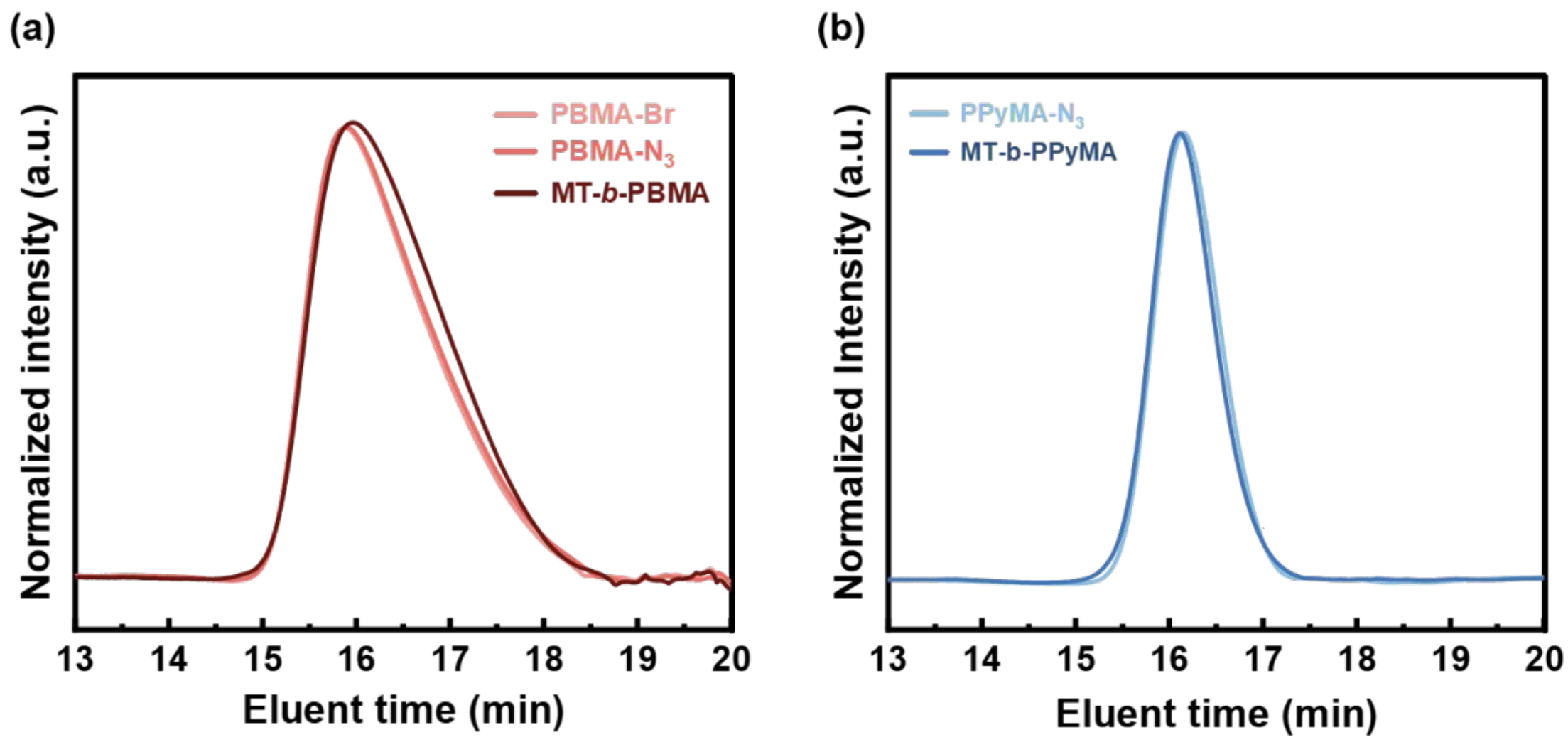

Figure S8. SEC traces of the polymer precursors and the final block copolymers. (a) PBMA-Br, PBMA-N_3, and MT-*b*-PBMA (M_n = 4,000 g/mol, $Đ$ = 1.29) using THF as eluent, and (b) PPyMA-N_3 and MT-*b*-PPyMA (M_n = 15,000 g/mol, $Đ$ = 1.21) using DMF as eluent.

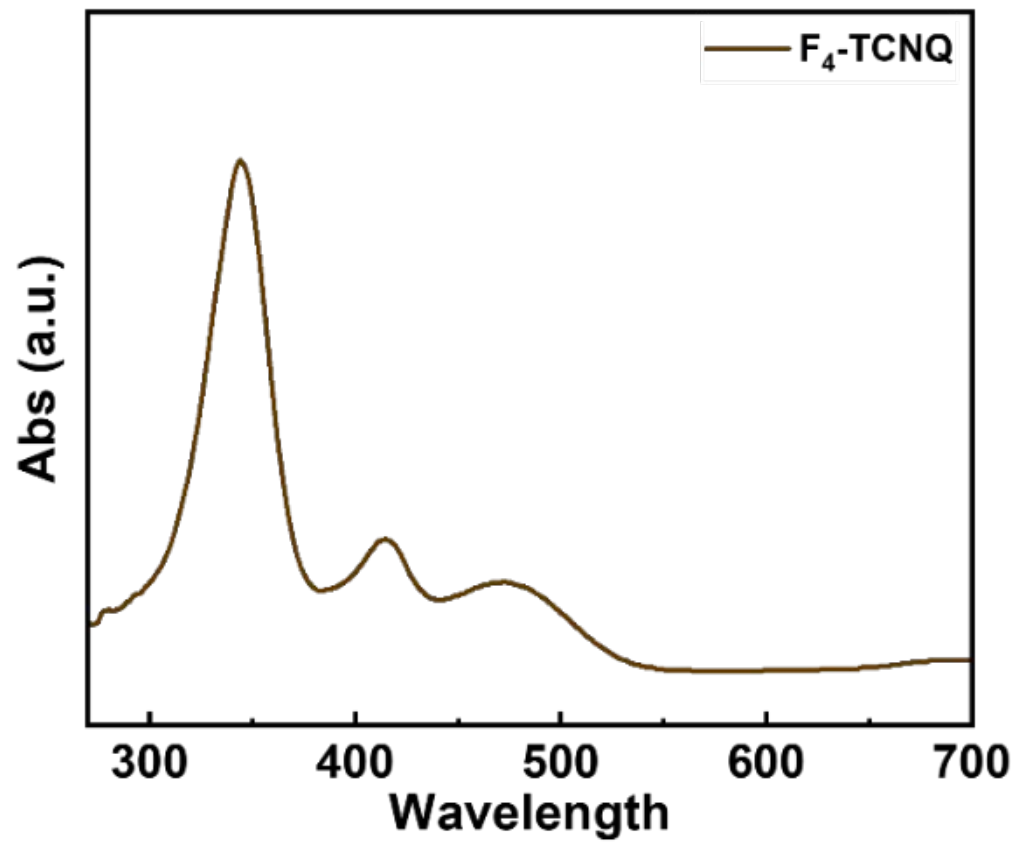

Figure S9. UV−vis optical absorption spectrum of F_4-TCNQ

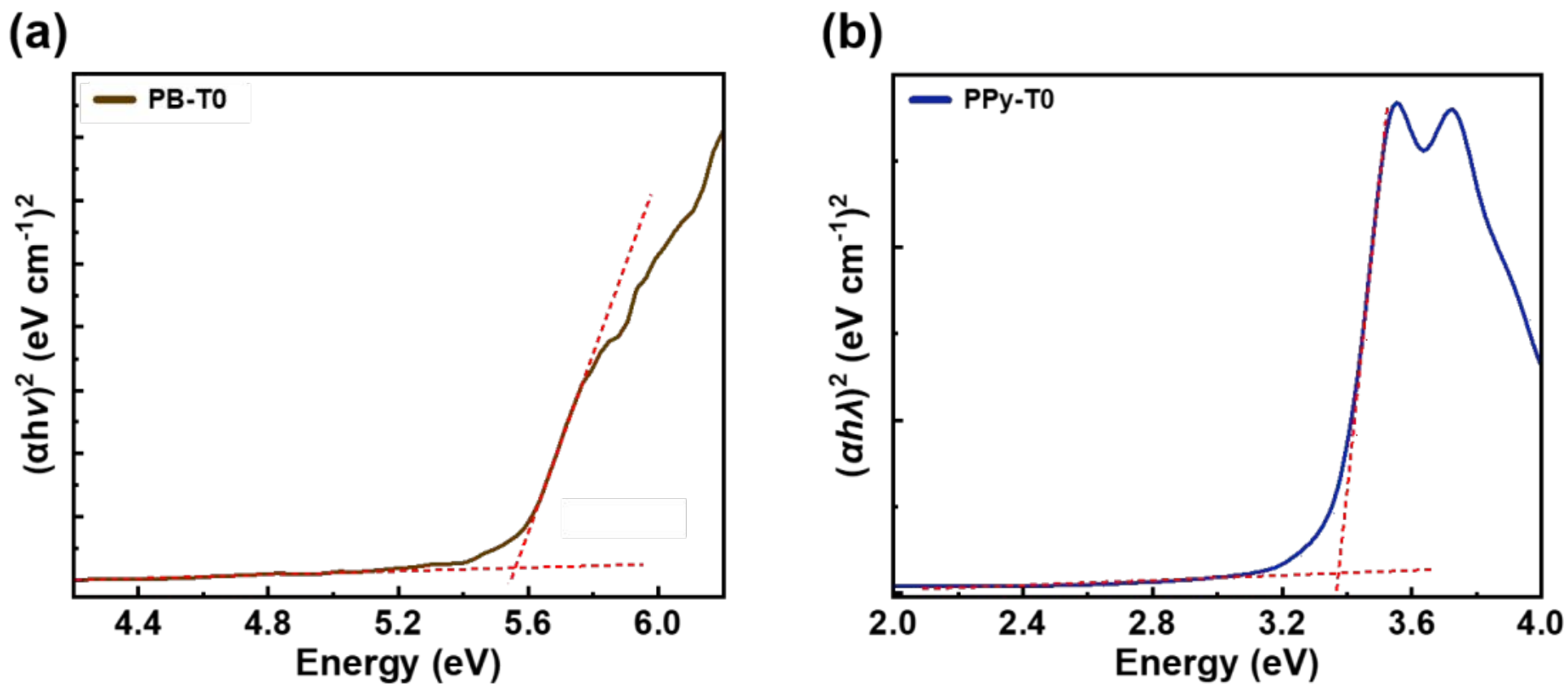

Figure S10. Tauc plots of (a) PB-T0 and (b) PPy-T0 without adding F_4-TCNQ.

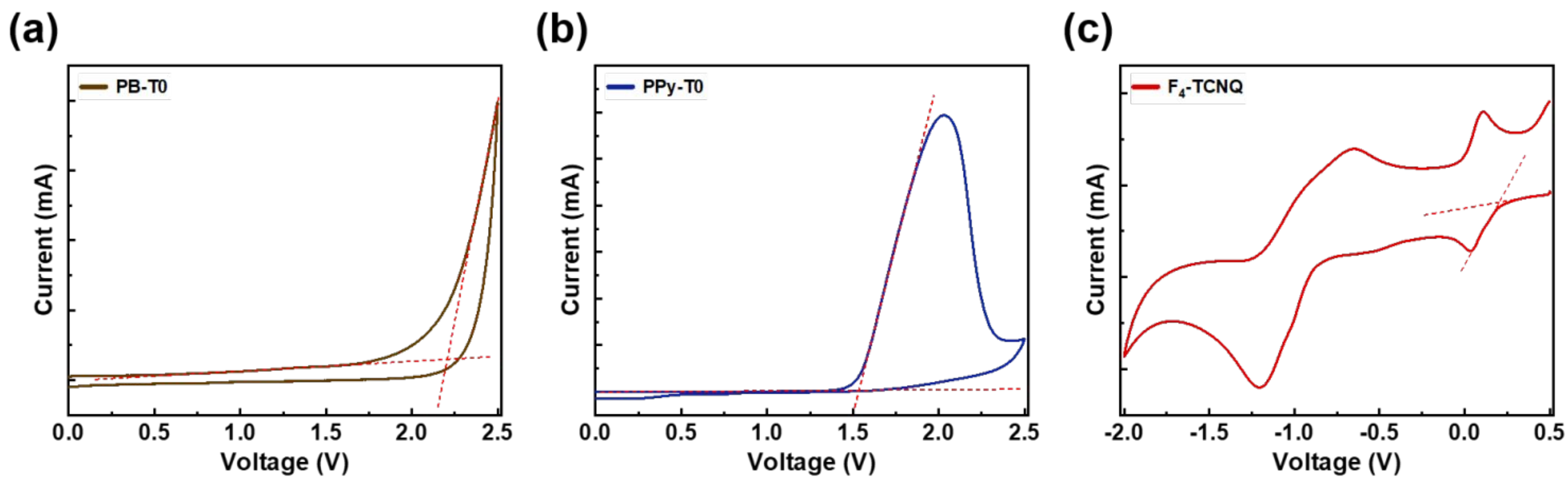

Figure S11. CV profiles of (a) PB-T0, (b) PPy-T0, and (c) F_4-TCNQ with Ag/AgCl as a reference electrode. The highest occupied molecular orbital (HOMO) is calculated using the equation: HOMO (eV) = $-e\left[E_{onset}^{OX} - E_{ferrocene}^{\frac{1}{2}} + 4.8\right]$. EOX onset and E1/2 ferrocene stand for the onset oxidation potentials of the materials and the reference redox couple of ferrocene.

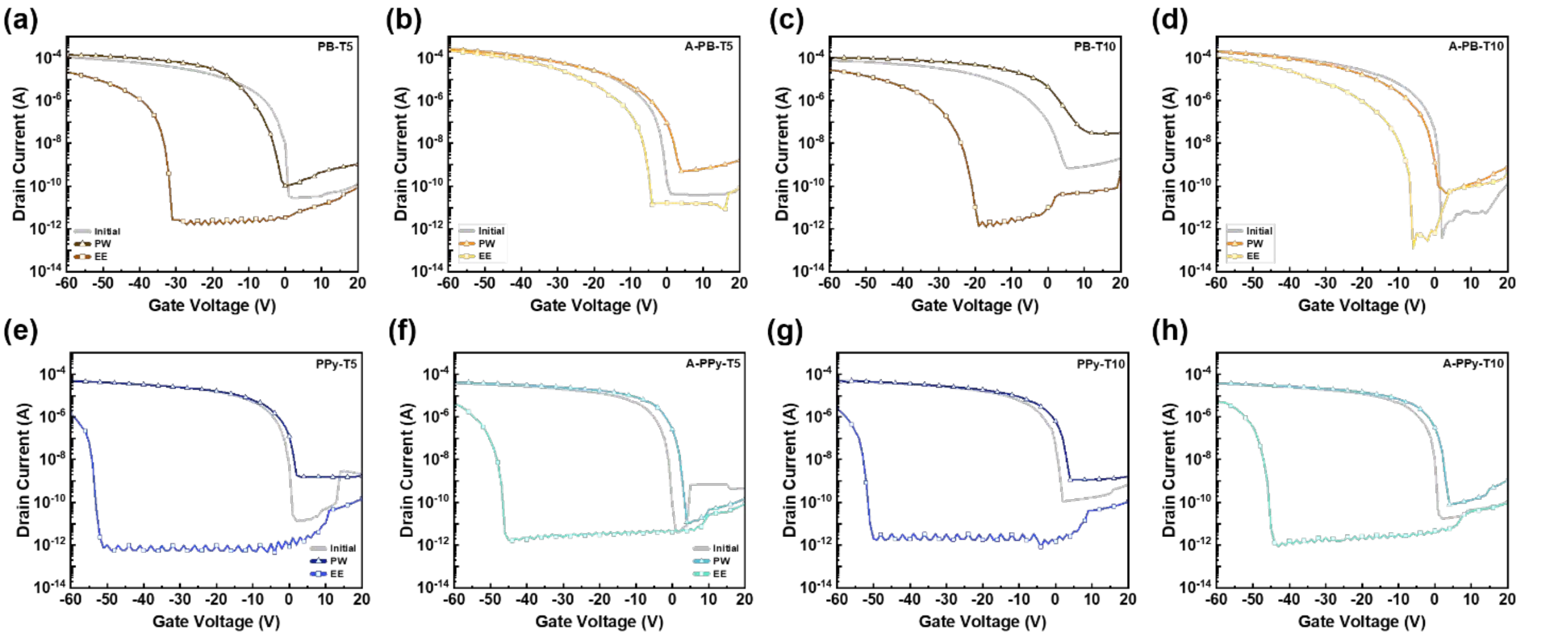

Figure S12. Transfer characteristics of (a) PB-T5, (b) A-PB-T5, (c) PB-T10, (d) A-PB-T10, (e) PPy-T5, (f) A-PPy-T5, (g) PPy-T10, and (h) A-PPy-T10. Note that the transfer curves were measured under V_d = –60 V and V_g from 20 to –60 V, and the photowriting process was performed under 365 nm illumination for 10 s at V_d = –60 V, whereas electrical erasing was conducted by applying V_g = –60 V for 1 s.

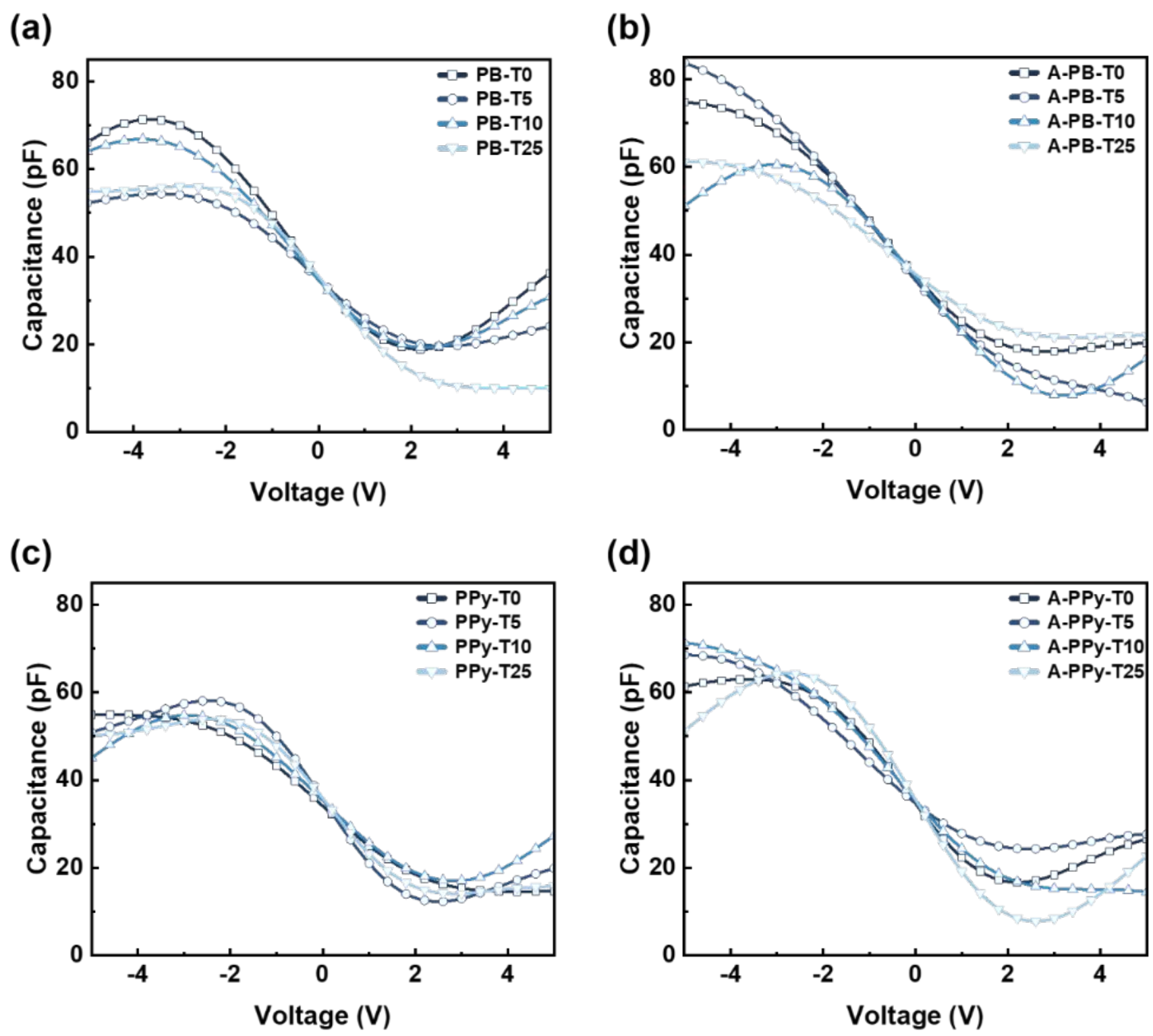

Figure S13. Capacitance–voltage (C–V) characteristics of (a) PB, (b) A-PB, (c) PPy, and (d) A-PPy supramolecular electrets. Note that the C–V measurements were performed using Au/electret/100 nm SiO_2/highly doped Si MIM capacitors with the applied bias swept from −5 to 5 V.

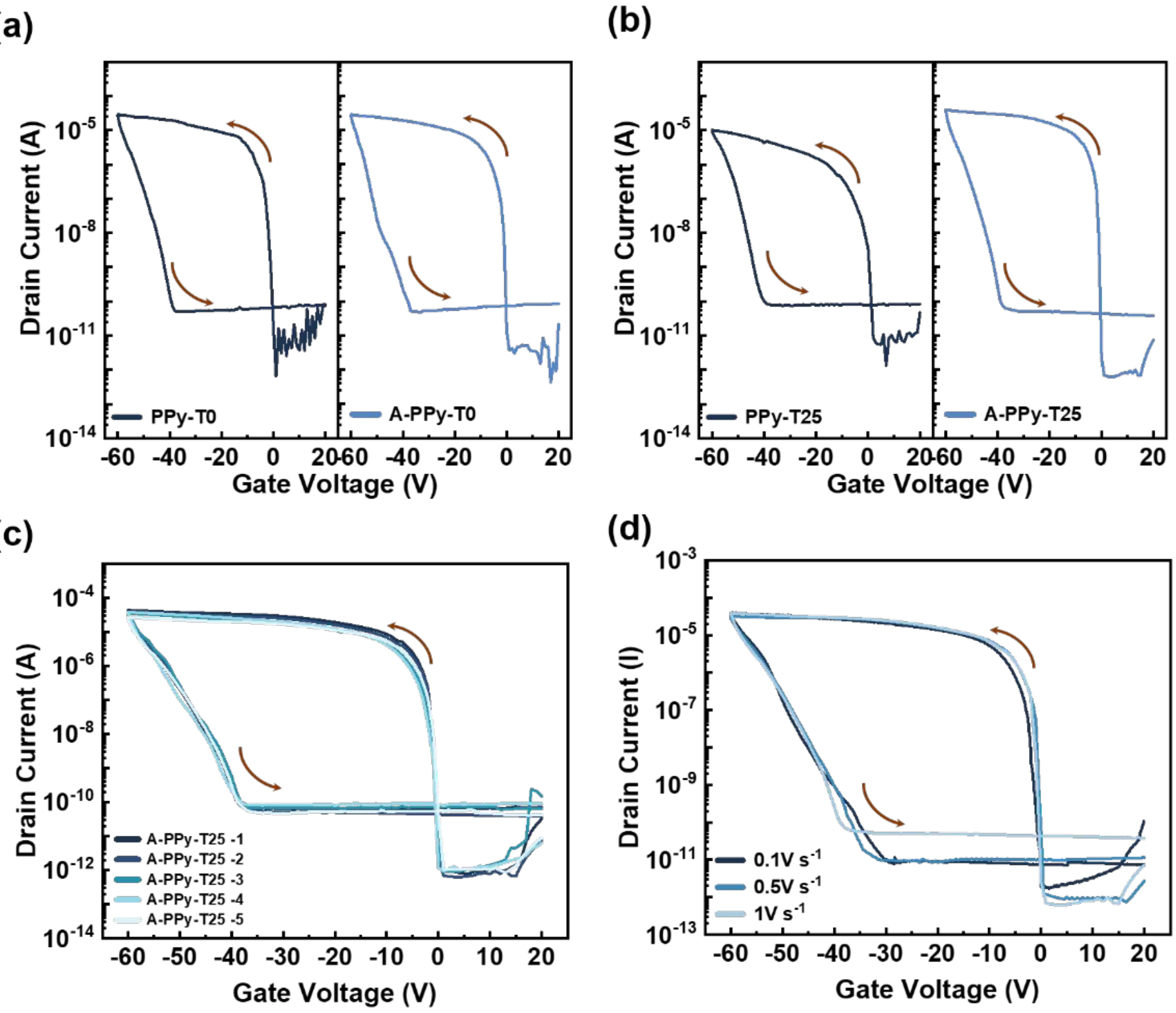

Figure S14. Dual-sweep transfer characteristics of (a) PPy-T0 and A-PPy-T0 and (b) PPy-T25 and A-PPy-T25. (c) Reproducibility of the optimized A-PPy-T25 device measured over five consecutive dual-sweep cycles using three independently fabricated devices. (d) Dual-sweep transfer characteristics of the A-PPy-T25 device measured at gate-voltage sweep rates of 0.1, 0.5, and 1 V s^{-1}. Note that the dual-sweep transfer characteristics were measured at V_d = –60 V with V_g swept from 20 to –60 V and then back to 20 V.

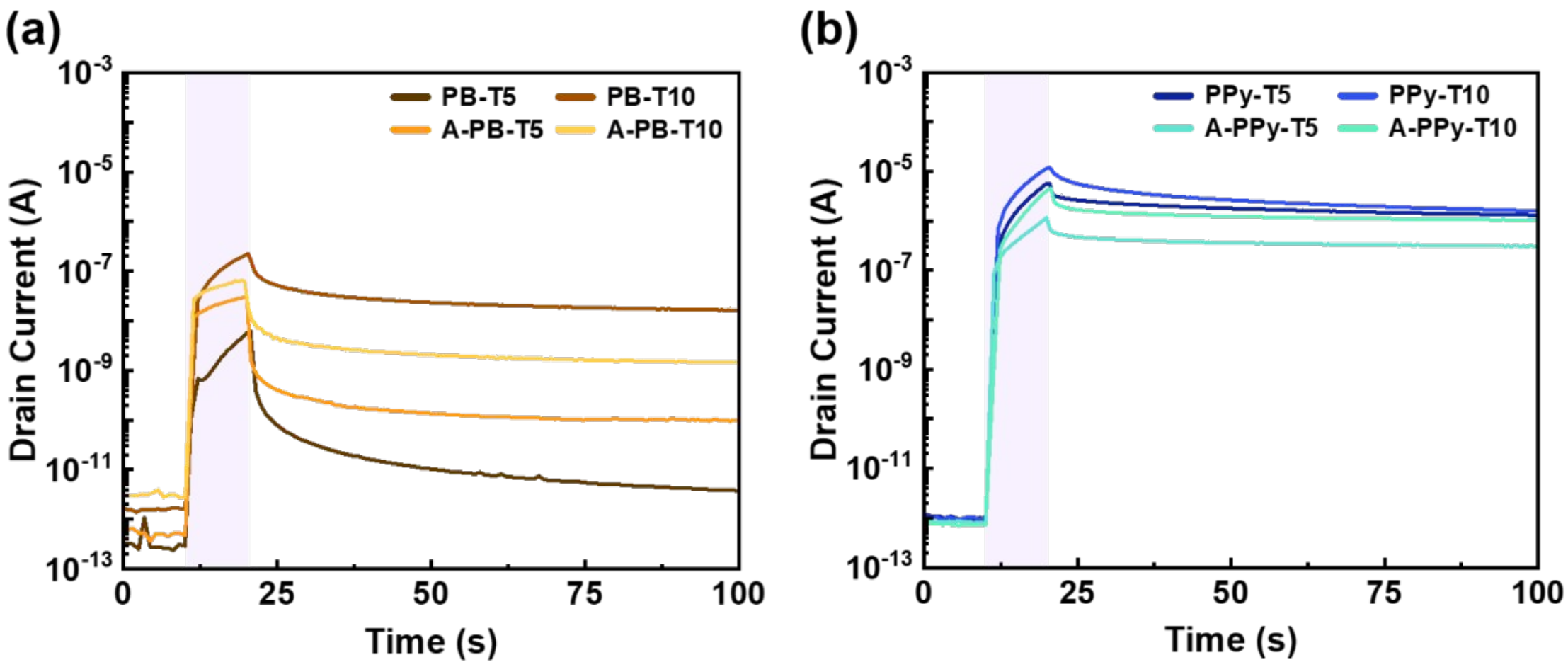

Figure S15. Transient characteristics of (a) PB and (b) PPy. Transient curves were measured at V_d = –60 V and V_g = 0 V, and photowriting was performed under 365-nm illumination for 10 s at V_d = –60 V, whereas electrical erasing was conducted by applying V_g = –60 V for 1 s.

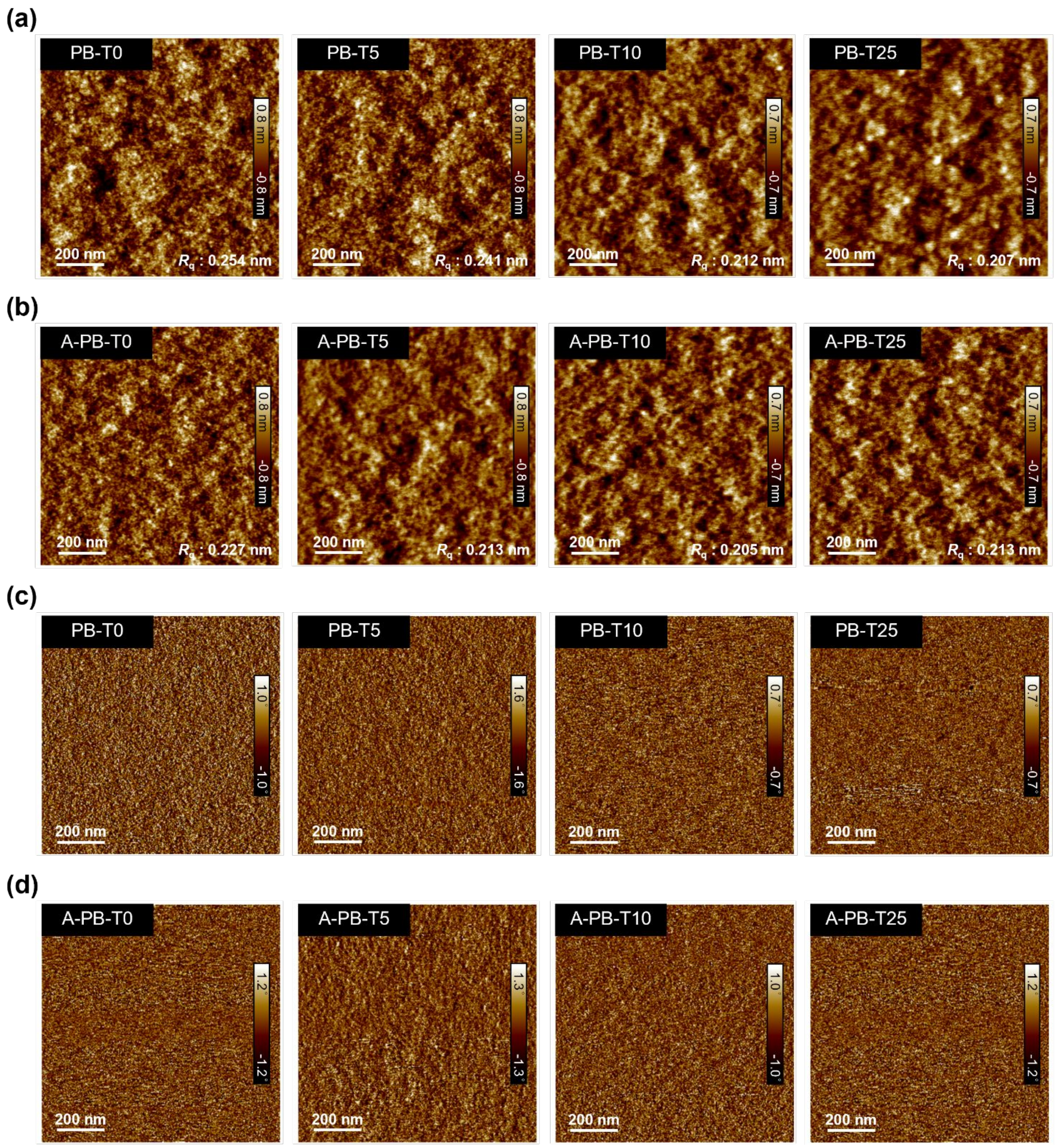

Figure S16. AFM (a,b) phase images and (c,d) height images of the PB-based electret (a,c) before (PB) and (b,d) after solvent vapor annealing (A-PB) with different F_4-TCNQ contents of 0–25 mol%.

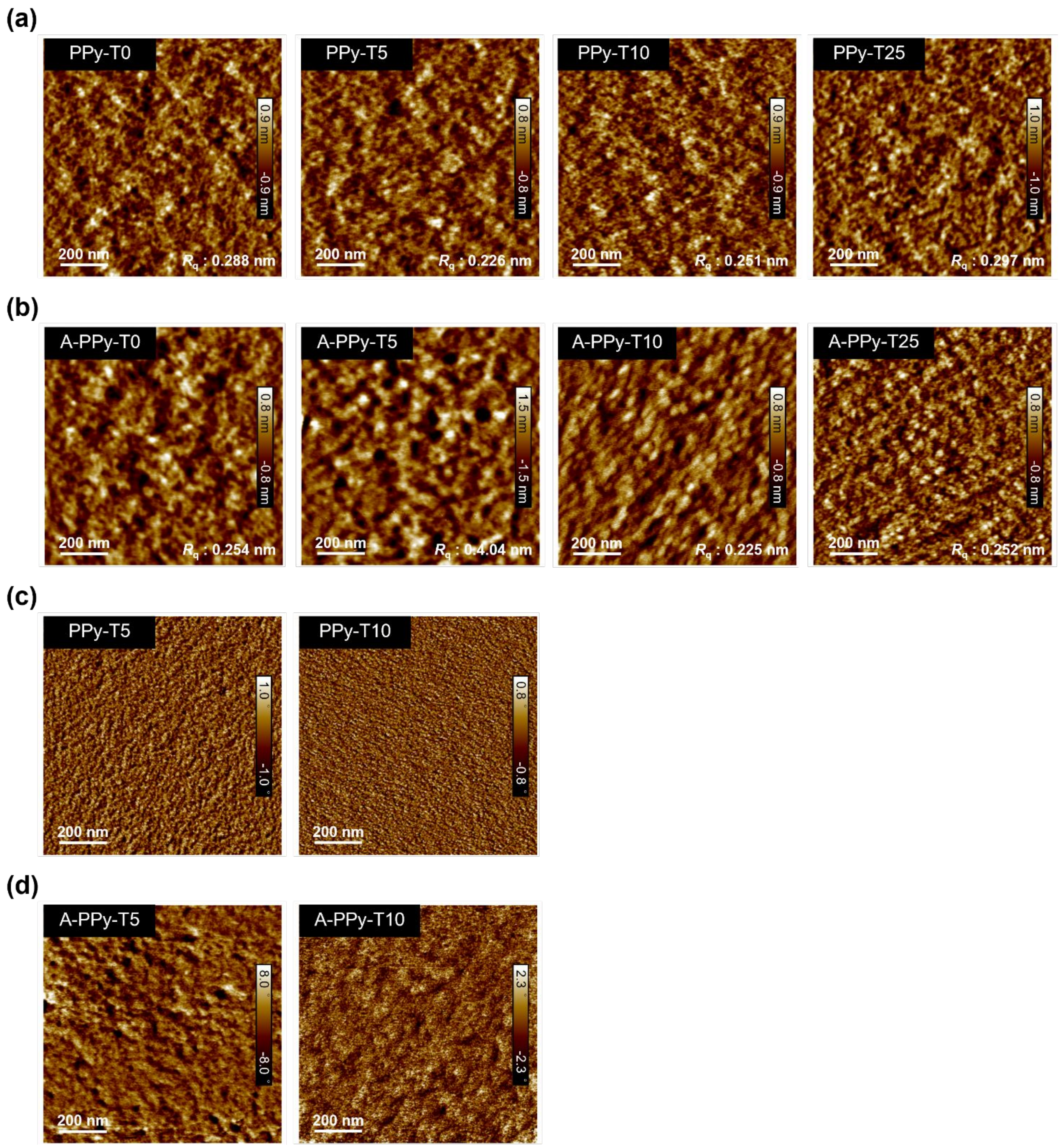

Figure S17. AFM (a,b) phase images and (c,d) height images of the PPy-based electret (a,c) before (PPy) and (b,d) after solvent vapor annealing (A-PPy) with different F_4-TCNQ contents of 0–25 mol%.

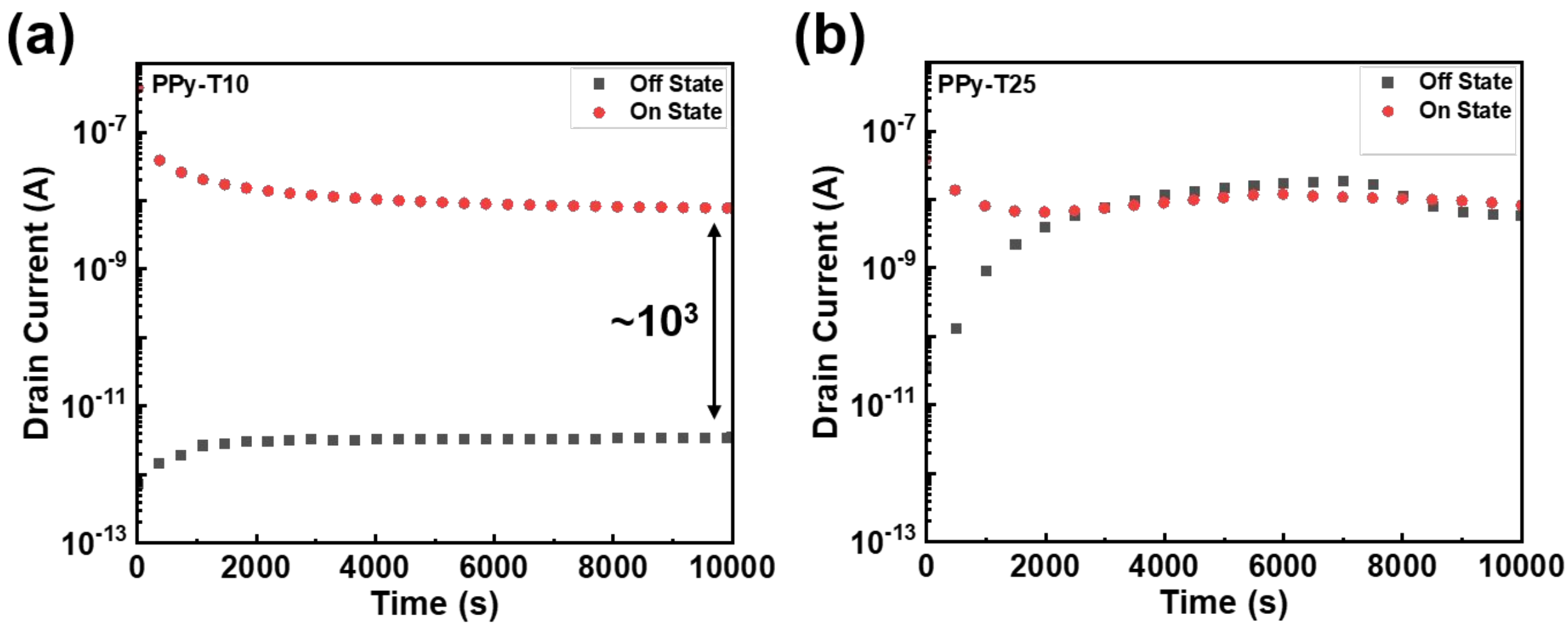

Figure S18. Memory retention characteristics of the phototransistor memory following photowriting and electrical erasing operations: (a) PPy-T10 and (b) PPy-T25 without solvent vapor annealing.

References in the Supporting Information

(S-1) Yang, W.-C.; Lin, Y.-C.; Liao, M.-Y.; Hsu, L.-C.; Lam, J.-Y.; Chuang, T.-H.; Li, G.-S.; Yang, Y.-F.; Chueh, C.-C.; Chen, W.-C. Comprehensive Nonvolatile Photoprogramming Transistor Memory via a Dual-Functional Perovskite-Based Floating Gate. *ACS Appl. Mater. Interfaces* **2021**, *13* (17), 20417–20426.

(S-2) Chang, Y. H.; Ku, C. W.; Zhang, Y. H.; Wang, H. C.; Chen, J. Y. Ultrafast Responsive Nonvolatile Flash Photomemory via Spatially Addressable Perovskite/Block Copolymer Composite Film. *Adv. Funct. Mater.* **2020**, *30* (21), 2000764.

(S-3) Park, B.-N. Nonvolatile C60 Self-Assembled Monolayer-Induced Photomemory Elements in Pentacene Organic Memory Field-Effect Transistors. *ACS Mater. Lett.* **2024**, *6* (7), 2526–2532.

(S-4) Ho, C.-H.; Lin, Y.-C.; Yang, W.-C.; Ercan, E.; Chiang, Y.-C.; Lin, B.-H.; Kuo, C.-C.; Chen, W.-C. Fast Photoresponsive Phototransistor Memory Using Star-Shaped Conjugated Rod–Coil Molecules as a Floating Gate. *ACS Appl. Mater. Interfaces* **2022**, *14* (13), 15468–15477.

(S-5) Shih, C.-C.; Chiang, Y.-C.; Hsieh, H.-C.; Lin, Y.-C.; Chen, W.-C. Multilevel Photonic Transistor Memory Devices Using Conjugated/Insulating Polymer Blend Electrets. *ACS Appl. Mater. Interfaces* **2019**, *11* (45), 42429–42437.

(S-6) Chen, Y. W.; Lin, Y. C.; Yang, W. C.; Yang, Y. F.; Peng, Y. C.; Chen, W. C.; Lin, B. H.; Yu, Y. Y.; Chen, W. C. Development of Multilevel Organic Phototransistor Memory Using Conjugated/Insulating Polymer Blends with Vertical Phase Separation. *Macromol. Mater. Eng.* **2022**, 307 (11), 2200388.

(S-7) Ke, C. Y.; Chen, M. N.; Chen, M. H.; Li, Y. T.; Chiu, Y. C.; Liou, G. S. Novel Authentic and Ultrafast Organic Photorecorders Enhanced by AIE-Active Polymer Electrets via Interlayer Charge Recombination. *Adv. Funct. Mater.* **2021**, *31* (28), 2101288.

(S-8) Weng, Y.-H.; Lin, Y.-C.; Ho, J.-C.; Yang, W.-C.; Lin, B.-H.; Liu, C.-L.; Chen, W.-C. High-Performance Phototransistor Memory with an Ultrahigh Memory Ratio Conferred Using Hydrogen-Bonded Supramolecular Electrets. *ACS Appl. Mater. Interfaces* **2023**, *15* (15), 19258–19269.

(S-9) Cheng, S.-W.; Han, T.; Huang, T.-Y.; Chang Chien, Y.-H.; Liu, C.-L.; Tang, B. Z.; Liou, G.-S. Novel Organic Phototransistor-Based Nonvolatile Memory Integrated with UV-Sensing/Green-Emissive Aggregation Enhanced Emission (AEE)-Active Aromatic Polyamide Electret Layer. *ACS Appl. Mater. Interfaces* **2018**, *10* (21), 18281–18288.
